# Comparing Effects of Biologic Agents in Treating Patients with Rheumatoid Arthritis: A Multiple Treatment Comparison Regression Analysis

**DOI:** 10.1371/journal.pone.0137258

**Published:** 2015-09-10

**Authors:** Ingunn Fride Tvete, Bent Natvig, Jørund Gåsemyr, Nils Meland, Marianne Røine, Marianne Klemp

**Affiliations:** 1 The Norwegian Computing Center, Oslo, Norway; 2 Department of Mathematics, University of Oslo, Oslo, Norway; 3 Smerud Medical Research International AS, Oslo, Norway; 4 Department of Pharmacology, University of Oslo, Oslo, Norway; 5 The Norwegian Knowledge Centre for the Health Services, Oslo, Norway; Nippon Medical School Graduate School of Medicine, JAPAN

## Abstract

Rheumatoid arthritis patients have been treated with disease modifying anti-rheumatic drugs (DMARDs) and the newer biologic drugs. We sought to compare and rank the biologics with respect to efficacy. We performed a literature search identifying 54 publications encompassing 9 biologics. We conducted a multiple treatment comparison regression analysis letting the number experiencing a 50% improvement on the ACR score be dependent upon dose level and disease duration for assessing the comparable relative effect between biologics and placebo or DMARD. The analysis embraced all treatment and comparator arms over all publications. Hence, all measured effects of any biologic agent contributed to the comparison of all biologic agents relative to each other either given alone or combined with DMARD. We found the drug effect to be dependent on dose level, but not on disease duration, and the impact of a high versus low dose level was the same for all drugs (higher doses indicated a higher frequency of ACR50 scores). The ranking of the drugs when given without DMARD was certolizumab (ranked highest), etanercept, tocilizumab/ abatacept and adalimumab. The ranking of the drugs when given with DMARD was certolizumab (ranked highest), tocilizumab, anakinra, rituximab, golimumab/ infliximab/ abatacept, adalimumab/ etanercept. Still, all drugs were effective. All biologic agents were effective compared to placebo, with certolizumab the most effective and adalimumab (without DMARD treatment) and adalimumab/ etanercept (combined with DMARD treatment) the least effective. The drugs were in general more effective, except for etanercept, when given together with DMARDs.

## Introduction

Disease modifying treatment has traditionally been given to rheumatoid arthritis (RA) patients at an early stage of the disease [1–2]. Early intervention could reduce joint damage and repress the RA progress [3]. For RA patients, biologic therapy has been a newer line of treatment typically following the firstly given disease modifying anti-rheumatic drugs (DMARDs). Biologic agents block certain chemicals in the blood from activating the immune system and hence protect patients’ joints [4]. A joint treatment regime of both biologic and DMARD therapy is recommended [5].

When assessing clinical efficacy of biologic agents most randomized controlled trials (RCTs) have compared the effect of one biologic agent versus placebo, either with or without additional DMARD treatment in both or one of the treatment arms. Few trials actively compared the effect of one biologic agent to another. Also, many trials contained multiple treatment arms which differ both with respect to dosing regime and endpoints measured. The great variety in comparisons and trial design has made it challenging to compare and rank biologic agents. We have taken a multiple treatment comparison (MTC) regression modelling approach. We have developed a model including all trials comparing the effect of a biologic drug against placebo or another biologic drug, with or without DMARD treatment. An advantage with MTCs is that one obtains treatment comparisons not directly observed. For example, with some trials comparing treatment one to treatment two and some trials comparing treatment two to treatment three there was an indirect comparison of treatment one to treatment three. Also, observed treatment comparisons could be strengthened from the indirect estimates. Hence, by taking this approach one could compare and rank all biologics with respect to their clinical effect.

Among the RA biologic agents on the market there have been five TNF inhibiting anti-inflammatory drugs (adalimumab, certolizumab, etanercept, golimumab, and infliximab), one interleukin-1 (IL-1) receptor antagonist (anakinra), one T-cell selective co-stimulation modulator (abatacept), one chimeric monoclonal CD20 antibody (rituximab), and one anti-IL-6 (tocilizumab).

Most systematic reviews compared some of these drugs, a few compared all of them, by including various trials reporting treatment effects [6]. A model approach to take into consideration whether patients have been given joint DMARD and biologic agent treatment or just a biologic agent alone has been called for [6]. This paper demonstrates such an approach by constructing a comprehensive statistical model, including both direct and indirect comparisons of the effect of all the nine biologic agents assessed in published trials. The objective of this paper was to compare and rank biologic agents’ clinical efficacy, when given alone or in combination with DMARDs in RA patients. We have developed a novel MTC regression analysis approach where we also take the disease duration and dose level into account.

## Materials and Methods

### Literature search

To identify published trials, we searched MEDLINE, Centre for Reviews and Dissemination, The Cochrane Library and the Drug Effectiveness Review Project’s website for systematic reviews assessing efficacy of biologic drugs in RA patients using included drugs (abatacept, adalimumab, anakinra, certolizumab, etanercept, golimumab, infliximab, rituximab and tocilizumab) and indication (rheumatoid arthritis) as search terms and mapped to MeSH (Medical Subject Headings) terminology. We attempted to identify additional trials through hand searches of reference lists of included trials and reviews. The search was last updated December 2014.

**Fig 1:**
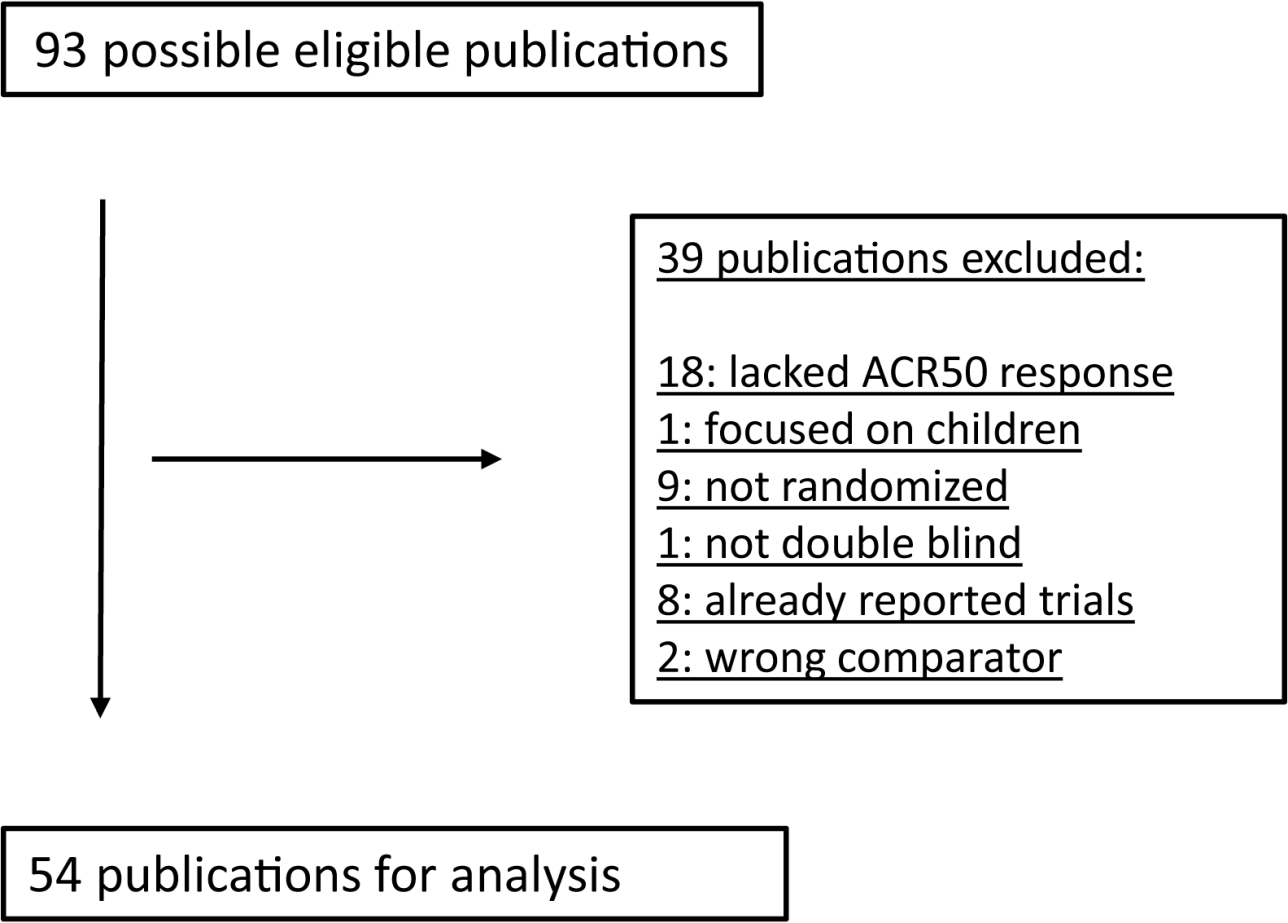
Flow chart. Study selection procedure.

Among the systematic reviews identified, we selected the most comprehensive and updated of high quality, which we used to identify 93 possible eligible publications. After closer inspection, we found that 39 publications were not relevant for our analysis and therefore excluded. We found that 18 lacked the ACR50 response, one focused only on children, nine were not randomized, one reported a trial which was not double blind, eight considered other already reported trials and two contained a comparison including NSAID treatment which we did not consider. The analysis was therefore based on 54 publications [7–60]. One of the 54 publications reported two different comparison groups [22], giving a total of 55 comparison groups. A list of the included publications with an overview of the different comparisons is presented in Table 1. Also see details of the study selection process in the flowchart in Fig 1 and Table A in S1 File. When considering publications reporting on the same trial at various time points we chose publications who reported trial duration closest to one year, see Table A in S1 File for details.

**Table 1:** The publications and the different treatment arms.

	Publication	Treat_arm 1_ *	Treat_arm 2_	Treat_arm 3_	Treat_arm 4_	Treat_arm 5_	Treat_arm 6_	Treat_arm 7_
1	Abe 2006	P+DM	INF+DM	INF+DM				
2	Emery 2008a	P+DM	TOC+DM	TOC+DM				
3	Genovese 2008a	DM	TOC+DM					
4	Maini 2006	P+DM	TOC	TOC	TOC	TOC+DM	TOC+DM	TOC+DM
5	Smolen 2008	P+DM	TOC+DM	TOC+DM				
6	Chen 2009	DM	ADA+DM					
7	Cohen 2002	P+DM	ANA+DM	ANA+DM	ANA+DM	ANA+DM	ANA+DM	
8	Cohen 2004	P+DM	ANA+DM					
9	Edwards 2004	DM	RIT+DM					
10	Emery 2006b	P+DM	RIT+DM	RIT+DM				
11	Emery 2009	P+DM	GOL+DM	GOL+DM				
12	Emery 2010	P+DM	RIT+DM	RIT+DM				
13	Fleischmann 2009	P	CER					
14	Kremer 2006	P+DM	ABA+DM					
15	Lipsky 2000	P+DM	INF+DM	INF+DM	INF+DM	INF+DM		
16	Maini 1999	P+DM	INF+DM	INF+DM	INF+DM	INF+DM		
17	Miyasaka 2008	P	ADA	ADA	ADA			
18	Moreland 1999	P	ETA	ETA				
19	Putte 2003	P	ADA	ADA	ADA			
20	Putte 2004	P	ADA	ADA	ADA	ADA		
21	Quinn 2005	P+DM	INF+DM					
22	Schiff 2008	P+DM	ABA+DM	INF+DM				
23	Schiff 2008	INF+DM	ABA+DM					
24	Nishimoto 2004	P	TOC	TOC				
25	Nishimoto 2007	DM	TOC					
26	Nishimoto 2009	P+DM	TOC+DM					
27	Smolen 2009	P+DM	CER+DM	CER+DM				
28	StClair 2004	P+DM	INF+DM	INF+DM				
29	Weinblatt 1999	P+DM	ETA+DM					
30	Weinblatt 2003	P+DM	ADA+DM	ADA+DM	ADA+DM			
31	Westhovens 2006b	P+DM	INF+DM	INF+DM				
32	Zhang 2006	P+DM	INF+DM					
33	Furst 2003	P	ADA					
34	Genovese 2005a	P	ABA					
35	Kay 2008	P+DM	GOL+DM					
36	Keystone 2004	P+DM	ADA+DM	ADA+DM				
37	Keystone 2008a	P+DM	CER+DM	CER+DM				
38	Kim 2007	P+DM	ADA+DM					
39	Klareskog 2004	P+DM	ETA+DM					
40	Kremer 2005	P+DM	ABA+DM	ABA+DM				
41	Keystone 2009a	P+DM	GOL+DM					
42	Cohen 2006	P+DM	RIT+DM					
43	Emery 2008b	P+DM	TOC+DM	TOC+DM				
44	Detert 2013	P+DM	ADA+DM					
45	Kavanaugh 2013	P+DM	ADA+DM					
46	Tak 2011	P+DM	RIT+DM	RIT+DM				
47	Choy 2012	P+DM	CER+DM					
48	Kremer 2010	P+DM	GOL+DM	GOL+DM				
49	Tanaka 2012	P+DM	GOL+DM	GOL+DM				
50	Kremer 2011	P+DM	TOC+DM	TOC+DM				
51	Jones 2010	P+DM	TOC					
52	Yazici 2012	P	TOC					
53	Breedveld 2006	DM	ADA+DM	ADA				
54	Westhovens 2009	P+DM	ABA+DM					
55	Weinblatt 2013	ADA+DM	ABA+DM					

*P. placebo, DM: DMARD, ADA: adalimumab, CER: certolizumab, ETA: etanercept, GOL: golimumab, INF: infliximab, ANA: anakinra, ABA: abatacept, RIT: rituximab, TOC: tocilizumab.

### Study selection

Two people independently reviewed full text of all the publications for inclusion. If both reviewers agreed that the reported trial did not meet eligibility criteria it was excluded. Records were considered for exclusion if they did not meet pre-established eligibility criteria with respect to trial design or duration, patient population, interventions, outcomes, and comparisons to medications outside our scope of interest. We considered adult patients diagnosed with RA who participated in a randomized, double blind clinical trial evaluating efficacy of biologic agents. We wanted not to distinguish between mode of administration (intravenous or oral) and to follow the intention to treat principle. We focused on the drugs’ effect and used the number of patients experiencing a 50% improvement according to the American College of Rheumatology (ACR) scale [61] (ACR50) as endpoint. Efficacy endpoints are the response ratios between biologic agent treated groups and placebo (with or without DMARD treatment). We take into consideration the influence that the disease duration at trial start and the dose level used can have on the treatment effect in scenario analyses.

#### Data

**Fig 2:**
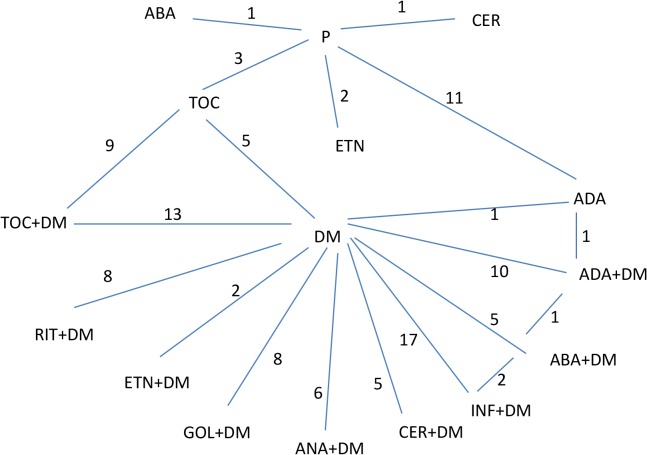
Direct and indirect comparisons. DM indicates either DMARD alone or placebo and DMARD treatment together.

Table 1 presents the trials entering the analysis, together with the drugs considered in each trial. For the number of participants and ACR50 responders in the trials, see the Table C in S1 File. We did not distinguish between patients given DMARD or DMARD plus placebo, since both groups were therapeutically comparable. Fig 2 displays the network of direct and indirect comparisons, where DM indicated either DMARD treatment alone or joint placebo and DMARD treatment. There were for example 11 group comparisons of ADA versus placebo treatment. In total there were 111 comparisons over 54 publications. All nine drugs were compared with placebo when given jointly with DMARD, but there were no comparisons of infliximab, anakinra, golimumab or rituximab without joint DMARD treatment.

The duration of RA was given as the average number of years of disease duration at trial start for each treatment arm, see Table D in S1 File. The dose levels were defined as either low or high, see Tables E and F in S1 File.

### Statistical analysis

We constructed a joint model for assessing the comparable relative effect between the various biologic drugs, placebo and DMARD for all trials, and performed a Bayesian statistical analysis based on this model inspired by Klemp et al. [62], now including regression terms taking the explanatory variables disease duration and dose level into consideration. The analysis embraced all treatment and comparator arms over the 54 publications. This way all measured effects of any biologic agent contributed to the comparison of all biologic agents relative to each other either alone or combined with DMARD.

In the following we let P and DM denote placebo and DMARD treatment respectively. Similarly, ADA: adalimumab, CER: certolizumab, ETN: etanercept, GOL: golimumab, INF: infliximab, ANA: anakinra, TOC: tocilizumab, ABA: abatacept and RIT: rituximab. The studies = 1, …, S, S = 55, had various numbers of arms, ranging from two to seven.

We present for simplicity first a model without explanatory variables and then include them. Let r_ij_ denote the number who achieves an ACR50 score in study i in arm j, i = 1, …, S, j = 1, …, a_i_, where a_i_ is the number of arms in study i, and a_i_ varies between two and seven arms. We let m_ij_ denote the number of patients in study i in arm j.

Let p_ki_ denote the probability in trial i to achieve an ACR50 score for treatment k, where k could be one of 16 different treatments: CER, ADA, ETN, TOC, ABA, DM/DM+P, INF+DM, ABA+DM, ANA+DM, CER+DM, GOL+DM, ADA+DM, TOC+DM, RIT+DM, ETN+DM and placebo (P). r_ij_ follows a binomial distribution. We assume that the multiplicative treatment effects relative to placebo are given by the effect-ratios *γ* ^*kp*^ = *p*_*ki*_/*p*_*pi*_, where k denotes treatment with CER, ADA, ETN, TOC, ABA, DM/DM+P,INF+DM, ABA+DM, ANA+DM, CER+DM, GOL+DM, ADA+DM, TOC+DM, RIT+DM and ETN+DM (k denotes now one of 15 different treatments, that is without placebo). We assume for now that the effect-ratios are constant over studies independent of explanatory variables.

Let *A*_*i*_ denote all treatment arms in study i. The likelihood is given by:

$$\prod_{i=1\;}^{S}\prod_{j\in A_{i}}\;{\left(p_{pi}\;\gamma^{\;j,p}\;\right)}^{r_{ij}}{\left(1\;-\;p_{pi}\;\gamma^{\;j,p}\;\right)}^{m_{ij}-\;r_{ij}}.$$

This Bayesian approach is hence based on the construction of probability distributions for the parameters to be estimated (p_pi_, the γs). This does not mean that these parameters should be interpreted as random variables, but our knowledge of the parameters is uncertain, and we describe this uncertainty through probability distributions.

Probability distributions describing our initial uncertainty are called prior distributions (that is, before the data are collected). When the study results are taken into account, the prior distributions are updated by Bayes’ formula to posterior distributions. With little information a priori we choose non-informative priors. Hence, we assume a priori that γ^j,p^_~_ normal(0,σ^2^_ϕ_), j = CER, …, ETN+DM and σ_ϕ_~uniform(0,20).

Parameters to be estimated in the model are the p_pi_s, γs and σ_ϕ_. We will assume a priori that p_pi_, i = 1, …, S, are uniformly distributed, with an upper limit dependent upon the γs in study i in the following way:

$$p_{pi}∼uniform\left(0,\text{min}\left(1,min_{j\in A_{i}}1/\gamma^{j,p}\right)\right).$$

Hence, the upper bound of the placebo response probabilities and medication response ratios constrain each other; a larger prior support for the placebo response probabilities (p_pi_) induces a smaller prior support for the medication response ratios (the γs). One must balance these two as shown in the equation above.

We now consider a model with explanatory variables. When we let the multiplicative treatment effects be dependent upon duration of disease (X^V^) and dose level (X^D^), where we let the impact of duration of disease and dose level be independent upon the given drug, we define

$$G_{ij}=\gamma^{jp}e^{\beta_{D}X_{ij}^{D}+\beta_{V}X_{ij}^{V}}.$$

The likelihood is now given by:

$$\prod_{i=1\;}^{S}\prod_{j\in A_{i}}\;{\left(p_{pi}\;G_{ij}\;\right)}^{r_{ij}}{\left(1\;-\;p_{pi}\;G_{ij}\;\right)}^{m_{ij}-\;r_{ij}}.$$

Now we assume a priori that

$$p_{pi}∼uniform\left(0,\text{min}\left(1,min_{j\in A_{i}}1/G_{ij}\right)\right).$$

The placebo-probabilities p_pi_ are hence assumed a priori to be uniformly distributed, in a range dependent upon disease duration and dose level in study i.

We let a priori β^V^ ~ normal(0,σ_V_^2^) and similarly β^D^ ~ normal(0,σ_D_^2^). The βs could be independent upon which TNF-inhibitor drug was given (giving one estimated β), as described above, or dependent giving one β for each of the TNF-inhibitor drugs. We let the a priori distributions for σ_V_ and σ_D_ both be uniformly distributed over (0,20).

This model was fitted in WinBUGS[63] run from R[64]. We did 500 000 burn-ins and thereafter 500 000 new updates where we took out every 200. This gave 2 500 samples from the full posterior conditional distribution for each of the parameters entering the model. Table A in S1 File contains the estimated parameters in the model.

We considered varieties of the model where the effect was dependent upon disease duration or dose level or both. The effect of disease duration and drug level could also be drug dependent or not. This resulted in eight model variations to be explored, in addition to one without any explanatory variables.

From the joint model, we estimated the relative effect of each biologic agent versus placebo (with or without DMARD treatment) and versus other biologic agents. As seen in Fig 2 biologic agents were given alone (without DMARD) for five drugs, while joint biologic and DMARD therapy was given for all the nine drugs included. There were 4*5/2 = 10 unique drug comparisons when given alone and 9*8/2 = 36 unique drug comparisons when given jointly with DMARD. Altogether this encompasses 46 comparisons of biologic agents against each other.

## Results

We included 54 publications for our MTC regression analysis, published between 1999 and 2013. Overall there were 19 798 patients given biologic treatment therapy, 1 165 given placebo and 8 037 given DMARD or joint DMARD and placebo treatment. The patient characteristic average disease duration for a treatment arm ranged from 0.13 to 13.1 years. Dose level was either low (in 51 arms) or high (in 48 arms). The trials lasted between 12 and 54 weeks, most of them lasting 24 weeks.

In the initial model fitting, the inclusion of explanatory variables did not alter the effect estimates of the agents much compared to not including them. Especially the ranking of the drugs relative to each other did not change. When we examined the impact of disease duration on treatment effect we found no significant effect. This was true both when the effect was drug dependent and when it was not. When we examined the impact of dose level on treatment effect we found that higher doses were associated with higher effect compared to lower doses (statistically significant), with a coefficient for dose level of 0.392, see Table B in S1 File. This was true only when the dose effect was drug independent.

When assuming an impact of both disease duration and dose level on treatment effect we found the effect of disease duration not to be statistically significant and the effect of dose level to be statistically significant when specified as drug independent. Hence, we concluded in our analysis that the disease duration had no impact on the drug effect but that dose level did, and the impact of a high versus low dose level was the same for all biologic drugs examined (in models with a drug dependent dose effect all dose parameters except the one for joint tocilizumab and DMARD treatment versus placebo were not significant).

We obtained two sets of results, one when the drugs were given alone and one when the drugs where given jointly with DMARD.

Table 2 displays the probability that one agent was better than another. The probability that certolizumab was better than etanercept when drugs were given alone was 0.69 while the probability that joint certolizumab and DMARD treatment was better than joint tocilizumab and DMARD treatment was 0.97 and so on. All drugs except etanercept had higher response ratios (drugs versus placebo) when taken jointly with DMARD.

**Table 2:** Probabilities that one agent was better than another, given alone (top) and together with DMARD (bottom).

**Biological agents given alone**								
	**ETN**	**TOC**	**ABA**	**ADA**				
**CER**	0.69	0.98	0.89	1				
**ETN**	-	0.72	0.75	0.98				
**TOC**	-	-	0.54	1				
**ABA**	-	-	-	0.87				
**Joint biological and DMARD therapy**								
	**TOC+DM**	**ANA+DM**	**RIT+DM**	**GOL+DM**	**INF+DM**	**ABA+DM**	**ADA+DM**	**ETN+DM**
**CER+DM**	0.97	0.98	1	1	1	1	1	1
**TOC+DM**	-	0.73	1	1	1	1	1	1
**ANA+DM**	-	-	0.88	0.96	0.98	0.99	1	1
**RIT+DM**	-	-	-	0.87	0.97	0.99	1	1
**GOL+DM**	-	-	-	-	0.62	0.72	0.9	0.94
**INF+DM**	-	-	-	-	-	0.7	0.94	0.96
**ABA+DM**	-	-	-	-	-	-	0.91	0.93
**ADA+DM**	-	-	-	-	-	-	-	0.76

**Fig 3:**
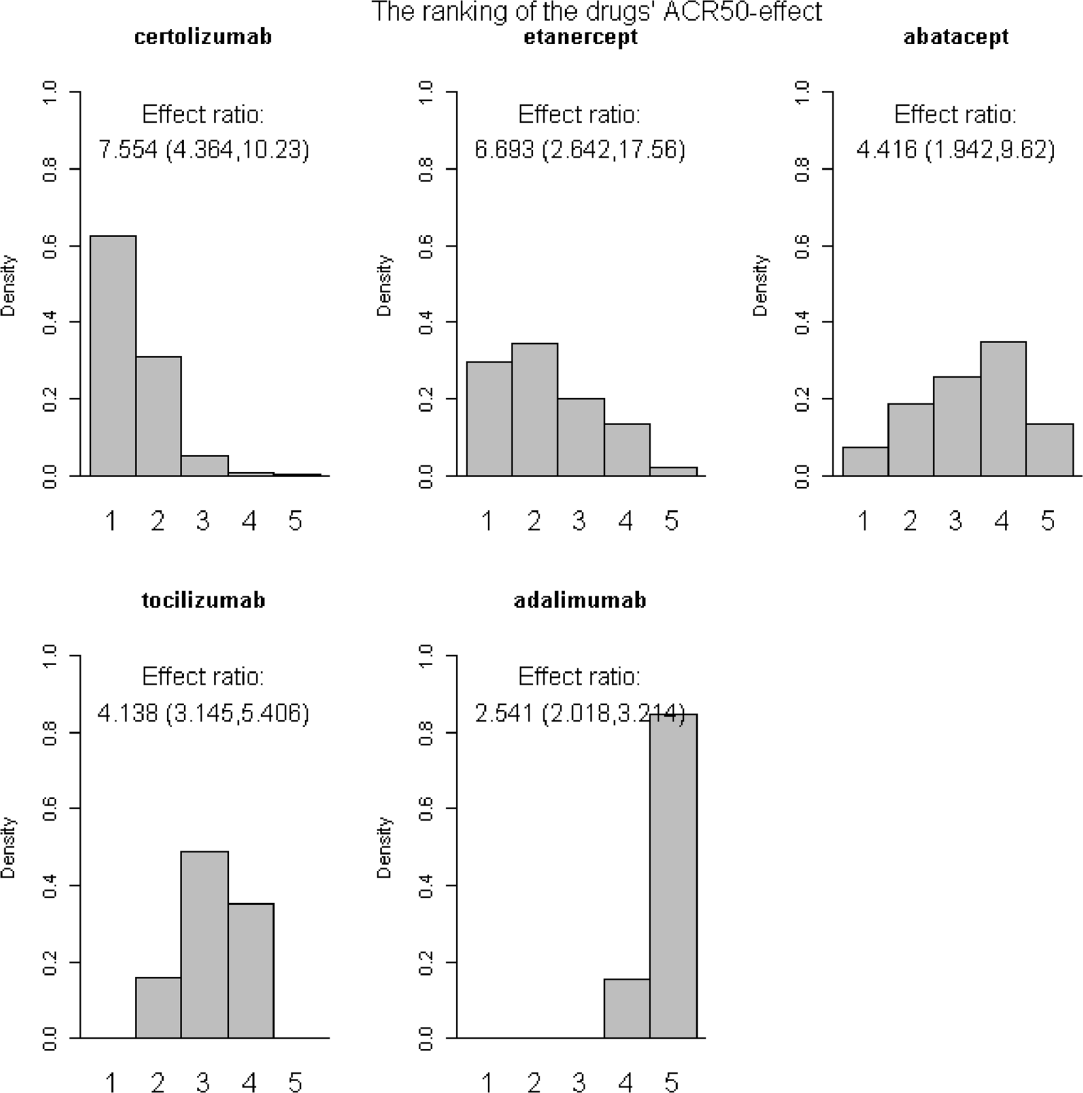
Histograms and ranking I. ACR50 effects as the response ratio versus placebo, drugs given alone.

**Fig 4:**
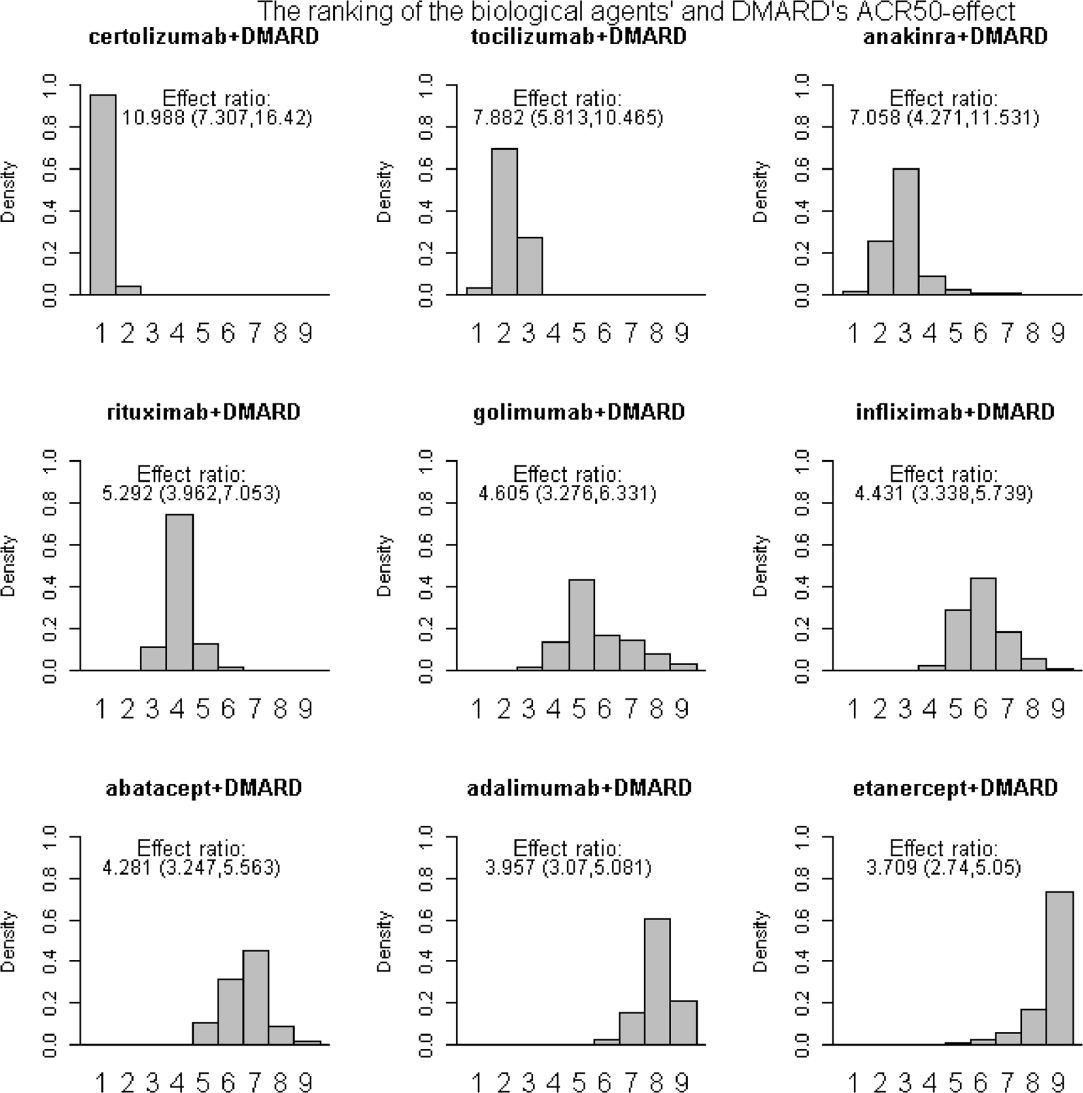
Histograms and ranking II. ACR50 effects as the response ratio versus placebo, drugs were given together with DMARD.

Based on the samples from the posterior distribution the agents were ranked according to the relative effect of drug versus placebo, giving histograms displaying the ranking (Fig 3 and 4). The height of the bars gives the probability of being ranked from position one to last. The effect ratios were the estimated effect of drug versus placebo treatment (Fig 3) or of joint drug and DMARD versus placebo treatment (Fig 4), see Table B in S1 File. Certolizumab was ranked as number one 62.8% of the time when given alone and 95.3% of the time when given with DMARD. Adalimumab was ranked as the least effective 84.4% of the times when given alone. Etanercept was 73.1% of the times ranked as the least effective when given with DMARD. The effect ratio of abatacept versus placebo treatment was 4.42 and the corresponding effect of tocilizumab was close in value, 4.14. Also, the probability that tocilizumab was better than abatacept was close to one half (0.54), indicating that these two drugs were equally effective.

The ranking of the drugs when given alone was, from most to least effective, certolizumab, etanercept, tocilizumab/ abatacept and adalimumab. All drugs were however effective, with response-ratios ranging from 2.54 (credibility interval 2.02 to 3.21) for adalimumab to 7.55 (credibility interval 4.36 to 10.23) for certolizumab.

The ranking of the drugs when given together with DMARD was, from most to least effective, certolizumab, tocilizumab, anakinra, rituximab, golimumab/ infliximab/ abatacept and adalimumab/etanercept. The effect ratio of joint golimumab and DMARD versus placebo treatment was 4.61 and the corresponding effects of joint infliximab and DMARD and joint abatacept and DMARD versus placebo treatment were 4.43 and 4.28 respectively. The probabilites that joint golimumab and DMARD treatment was better than joint infliximab and DMARD and joint abatacept and DMARD treatment were 0.62 and 0.72 respectively. The probability that joint infliximab and DMARD treatment was better than joint abatacept and DMARD treatment was 0.7. Hence, one could argue that these three drugs were not that different with respect to effect.

The effect ratio of joint adalimumab and DMARD versus placebo treatment was 4.00 and the corresponding effect of joint etanercept and DMARD treatment was 3.71. Also, the probability that joint adalimumab and DMARD treatment was better than joint etanercept and DMARD treatment was 0.76. These two treatments also seem to be similar with respect to effect. All agents were effective when given together with DMARD, with response-ratios ranging from 3.71 (credibility interval 2.74 to 5.05) for etanercept to 10.99 (credibility interval 7.31 to 16.42) for certolizumab.

The ranking of drugs given jointly with DMARD was for certolizumab, abatacept and adalimumab the same as the ranking when drugs were given without DMARD treatment. Etanercept was on the other hand ranked higher and tocilizumab lower when given alone compared to given with DMARD treatment. Tocilizumab had an almost twice as high response ratio while the effect of etanercept was almost halved when given with DMARD. Hence, unlike the other drugs, etanercept had a much higher effect when given alone than with joint DMARD treatment, but the uncertainty concerning the effect of exclusive etanercept treatment was very large.

## Discussion

All biological agents were effective compared to placebo, with certolizumab the most effective. The drugs were in general more effective when given together with DMARDs which was in harmony with current guidelines [5]. The dose level influenced the treatment effect (statistically significant), with a higher dose indicating a larger effect. We found no significant difference between longer versus shorter disease duration on the treatment effect. Higher doses could of course also lead to more adverse effects, an issue not focused on in this paper, and our finding should be interpreted with this in mind. Still, as various studies and even treatment arms had different dosing regiments it was important to correct for it in the analysis, giving estimated relative drug effects not confounded by dose level.

The way patients have been included in the trials and treated throughout the trial period could have changed over the publication years 1999 through 2013, possibly biasing the results. Clearly this would be a problem if some drugs were studied in earlier years and others in later years. This was not the case. Also, we found no apparent time trends regarding disease duration. The variation in trial duration could also potentially bias the ranking of the treatments. However, the different biologic agents were reasonably similarly distributed with respect to trial duration, with trials involving anakinra as perhaps an exception, all lasting only 24 or 26 weeks.

Previous MTC analyses of clinical trials reporting effects of biologic drugs indicated different rankings of the drugs [6], and this analysis has been an additional contribution.

For example, Nixon et al. [65] compared the effect of etanercept, infliximab, adalimumab and anakinra in 13 publications, none with head-to-head comparisons. Different models gave varying degrees of efficacy. In a final model they found anakinra to be the least effective. Singh et al. [66] compared abatacept, adalimumab, etanercept, infliximab, rituximab and anakinra from 27 clinical trials, none contained head-to-head comparisons. They reported a low effect of anakinra based on the results from three trials only. Divine et al. [67] and Turkstra et al. [68] both compared all nine biologic drugs and also ranked anakinra as the least effective drug. However, in all the mentioned publications there was little evidence of statistically significant differences in effects between anakinra and the other biologic drugs. In our analysis, anakinra (together with DMARD treatment) was ranked as number three. Golimumab was also ranked as relatively low in effect in both Divine et al. [67] and Mandema et al. [69], while this study ranked it somewhat higher.

Thorlund et al. [6] stated: “*Since future RA MTCs will undoubtedly continue to include both monotherapy and combination therapy trials*, *it is important to establish whether the concomitant use of DMARDs does in fact yield an effect modification on the relative comparative treatment effects*. *This will require more refined analyses than what have currently been performed among the MTCs”*. Our approach answers this request by distinguishing between joint DMARD or exclusively biologic drug use, and by letting this difference be drug dependent. And we find that for especially etanercept treatment this distinction truly matters. Our comparison network (Fig 2) resembles the one suggested in Thorlund et al. [6].

An advantage of our approach was the capability to include results from many trials into one model simultaneously, whether the trials compared biologic drugs alone or with DMARD treatment. In previous MTC-analyses only Mandema et al. [69] reported many trials (50), but it was unclear how many went into the analysis of ranking the drugs with respect to the ACR50 score. Our analysis included both direct and indirect comparisons from 54 publications in the same network, and we interpreted the results by probabilities tied to comparisons and ranking of the drugs. This approach could be particularly useful in situations when new drugs are introduced to establish a possible therapeutic benefit compared to already existing drugs.

A possible weakness of any such MTC analysis is that the trials combined might differ with respect to patient characteristics, follow-up time or other trial procedures, potentially introducing heterogeneity in the treatment effects. An MTC regression analysis will try to capture some of the possible differences, and we have considered the impact of dose level and disease duration in this analysis. Other explanatory variables could also have been considered, as geographical information on trial location or more detailed information on previous patient history. For some trials this could be available information, but not always. Correcting for many factors could exclude studies due to lack of information, introducing selection bias.

The drugs were ranked with respect to efficacy. We did not consider adverse events. Of course, adverse events are also important. This was however a more complicated task as the publications reported adverse events differently. Also, some publications did not report adverse events at all.

With many MTC analyses within the RA field published over the last five years with slightly different results it is clear that ranking these drugs has been challenging. The final result would depend on the chosen model, which trials are included, how heterogeneity is handled, if and which explanatory variables are taken into consideration and if and how joint treatment with other drugs are considered. We believe that our unified approach of including all relevant comparisons, both direct and indirect comparisons that exist, while specifying whether the drugs were given alone or in combination with DMARDs, is a sensible approach giving the possibility to rank all biologic drugs with respect to comparative effectiveness.

## Supporting Information

S1 PRISMA Checklist (DOC)

S1 File (DOCX)
