# Correction: Comparing Effects of Biologic Agents in Treating Patients with Rheumatoid Arthritis: A Multiple Treatment Comparison Regression Analysis

**DOI:** 10.1371/journal.pone.0146633

**Published:** 2016-01-26

**Authors:** Ingunn Fride Tvete, Bent Natvig, Jørund Gåsemyr, Nils Meland, Marianne Røine, Marianne Klemp

An error occurred during the revision process for this article. While re-running the model, the last treatment-arm in study number 7 (Cohen 2002), considering 5 treatment arms with anarkinra + DMARD versus DMARD (+ placebo) became wrongfully linked up to the treatment effect of certolizumab + DMARD rather than anarkinra + DMARD. As a result, the treatment effect of anarkinra + DMARD was estimated to be somewhat high and the treatment effect of certolizumab + DMARD somewhat lower. The treatment effect of certolizumab + DMARD should be 12.989 (and not 10.988 as reported in the article) and the treatment effect of anarkinra + DMARD should be reported as 5.767 (and not 7.058 as reported in the article). The other treatment effects are either unadjusted or adjusted with a low decimal.

The order of the drugs with respect to effect is not altered. Due to the now lower estimated effect of anarkinra + DMARD, it should be ranked as equally effective as rituximab + DMARD, so that the ranking of drugs when given with DMARD is, from most to least effective, certolizumab, tocilizumab, anakinra/ rituximab, golimumab/ infliximab/ abatacept and adalimumab/etanercept.

With respect to these changes, the eight sentence of the Abstract should read: The ranking of the drugs when given with DMARD was certolizumab (ranked highest), tocilizumab, anakinra/rituximab, golimumab/ infliximab/ abatacept, adalimumab/ etanercept.

Please see the corrected Results section below, as well as the corrected Table 2, Fig 3, Fig 4, and S1 File. In addition, “ETA” in the caption of Table 1 should read “ETN.” Please see the corrected Table 1 caption here.

**Table 1:** The publications and the different treatment arms.

	Publication	Treat_arm 1_*	Treat_arm 2_	Treat_arm 3_	Treat_arm 4_	Treat_arm 5_	Treat_arm 6_	Treat_arm 7_
1	Abe 2006	P+DM	INF+DM	INF+DM				
2	Emery 2008a	P+DM	TOC+DM	TOC+DM				
3	Genovese 2008a	DM	TOC+DM					
4	Maini 2006	P+DM	TOC	TOC	TOC	TOC+DM	TOC+DM	TOC+DM
5	Smolen 2008	P+DM	TOC+DM	TOC+DM				
6	Chen 2009	DM	ADA+DM					
7	Cohen 2002	P+DM	ANA+DM	ANA+DM	ANA+DM	ANA+DM	ANA+DM	
8	Cohen 2004	P+DM	ANA+DM					
9	Edwards 2004	DM	RIT+DM					
10	Emery 2006b	P+DM	RIT+DM	RIT+DM				
11	Emery 2009	P+DM	GOL+DM	GOL+DM				
12	Emery 2010	P+DM	RIT+DM	RIT+DM				
13	Fleischmann 2009	P	CER					
14	Kremer 2006	P+DM	ABA+DM					
15	Lipsky 2000	P+DM	INF+DM	INF+DM	INF+DM	INF+DM		
16	Maini 1999	P+DM	INF+DM	INF+DM	INF+DM	INF+DM		
17	Miyasaka 2008	P	ADA	ADA	ADA			
18	Moreland 1999	P	ETN	ETN				
19	Putte 2003	P	ADA	ADA	ADA			
20	Putte 2004	P	ADA	ADA	ADA	ADA		
21	Quinn 2005	P+DM	INF+DM					
22	Schiff 2008	P+DM	ABA+DM	INF+DM				
23	Schiff 2008	INF+DM	ABA+DM					
24	Nishimoto 2004	P	TOC	TOC				
25	Nishimoto 2007	DM	TOC					
26	Nishimoto 2009	P+DM	TOC+DM					
27	Smolen 2009	P+DM	CER+DM	CER+DM				
28	StClair 2004	P+DM	INF+DM	INF+DM				
29	Weinblatt 1999	P+DM	ETN+DM					
30	Weinblatt 2003	P+DM	ADA+DM	ADA+DM	ADA+DM			
31	Westhovens 2006b	P+DM	INF+DM	INF+DM				
32	Zhang 2006	P+DM	INF+DM					
33	Furst 2003	P	ADA					
34	Genovese 2005a	P	ABA					
35	Kay 2008	P+DM	GOL+DM					
36	Keystone 2004	P+DM	ADA+DM	ADA+DM				
37	Keystone 2008a	P+DM	CER+DM	CER+DM				
38	Kim 2007	P+DM	ADA+DM					
39	Klareskog 2004	P+DM	ETN+DM					
40	Kremer 2005	P+DM	ABA+DM	ABA+DM				
41	Keystone 2009a	P+DM	GOL+DM					
42	Cohen 2006	P+DM	RIT+DM					
43	Emery 2008b	P+DM	TOC+DM	TOC+DM				
44	Detert 2013	P+DM	ADA+DM					
45	Kavanaugh 2013	P+DM	ADA+DM					
46	Tak 2011	P+DM	RIT+DM	RIT+DM				
47	Choy 2012	P+DM	CER+DM					
48	Kremer 2010	P+DM	GOL+DM	GOL+DM				
49	Tanaka 2012	P+DM	GOL+DM	GOL+DM				
50	Kremer 2011	P+DM	TOC+DM	TOC+DM				
51	Jones 2010	P+DM	TOC					
52	Yazici 2012	P	TOC					
53	Breedveld 2006	DM	ADA+DM	ADA				
54	Westhovens 2009	P+DM	ABA+DM					
55	Weinblatt 2013	ADA+DM	ABA+DM					

*P. placebo, DM: DMARD, ADA: adalimumab, CER: certolizumab, ETN: etanercept, GOL: golimumab, INF: infliximab, ANA: anakinra, ABA: abatacept, RIT: rituximab, TOC: tocilizumab

**Results**

We included 54 publications for our MTC regression analysis, published between 1999 and 2013. Overall there were 19 798 patients given biologic treatment therapy, 1 165 given placebo and 8 037 given DMARD or joint DMARD and placebo treatment. The patient characteristic average disease duration for a treatment arm ranged from 0.13 to 13.1 years. Dose level was either low (in 51 arms) or high (in 48 arms). The trials lasted between 12 and 54 weeks, most of them lasting 24 weeks.

In the initial model fitting, the inclusion of explanatory variables did not alter the effect estimates of the agents much compared to not including them. Especially the ranking of the drugs relative to each other did not change. When we examined the impact of disease duration on treatment effect we found no significant effect. This was true both when the effect was drug dependent and when it was not. When we examined the impact of dose level on treatment effect we found that higher doses were associated with higher effect compared to lower doses (statistically significant), with a coefficient for dose level of 0.39, see Table B in S1 File. This was true only when the dose effect was drug independent.

When assuming an impact of both disease duration and dose level on treatment effect we found the effect of disease duration not to be statistically significant and the effect of dose level to be statistically significant when specified as drug independent. Hence, we concluded in our analysis that the disease duration had no impact on the drug effect but that dose level did, and the impact of a high versus low dose level was the same for all biologic drugs examined (in models with a drug dependent dose effect all dose parameters except the one for joint tocilizumab and DMARD treatment versus placebo were not significant).

We obtained two sets of results, one when the drugs were given alone and one when the drugs where given jointly with DMARD.

Table 2 displays the probability that one agent was better than another. The probability that certolizumab was better than etanercept when drugs were given alone was 0.71 while the probability that joint certolizumab and DMARD treatment was better than joint tocilizumab and DMARD treatment was 0.97 and so on. All drugs except etanercept had higher response ratios (drugs versus placebo) when taken jointly with DMARD.

**Table 2:** Probabilities that one agent was better than another, given alone (top) and together with DMARD (bottom).

**Biological agents given alone**								
	**ETN**	**TOC**	**ABA**	**ADA**				
**CER**	0.71	0.97	0.88	1				
**ETN**	-	0.73	0.72	0.98				
**TOC**	-	-	0.55	1				
**ABA**	-	-	-	0.86				
**Joint biological and DMARD therapy**								
	**TOC+DM**	**ANA+DM**	**RIT+DM**	**GOL+DM**	**INF+DM**	**ABA+DM**	**ADA+DM**	**ETN+DM**
**CER+DM**	0.99	1	1	1	1	1	1	1
**TOC+DM**	-	0.92	1	1	1	1	1	1
**ANA+DM**	-	-	0.57	0.78	0.83	0.87	0.93	0.95
**RIT+DM**	-	-	-	0.88	0.97	0.99	1	1
**GOL+DM**	-	-	-	-	0.59	0.7	0.9	0.94
**INF+DM**	-	-	-	-	-	0.69	0.95	0.96
**ABA+DM**	-	-	-	-	-	-	0.91	0.93
**ADA+DM**	-	-	-	-	-	-	-	0.76

Based on the samples from the posterior distribution the agents were ranked according to the relative effect of drug versus placebo, giving histograms displaying the ranking (Figs 3 and 4). The height of the bars gives the probability of being ranked from position one to last. The effect ratios were the estimated effect of drug versus placebo treatment (Fig 3) or of joint drug and DMARD versus placebo treatment (Fig 4), see Table B in S1 File. Certolizumab was ranked as number one 64.7% of the time when given alone and 98.76% of the time when given with DMARD. Adalimumab was ranked as the least effective 84.1% of the times when given alone. Etanercept was 71.5% of the times ranked as the least effective when given with DMARD. The effect ratio of abatacept versus placebo treatment was 4.41 and the corresponding effect of tocilizumab was close in value, 4.17. Also, the probability that tocilizumab was better than abatacept was close to one half (0.55), indicating that these two drugs were equally effective.

**Fig 3:**
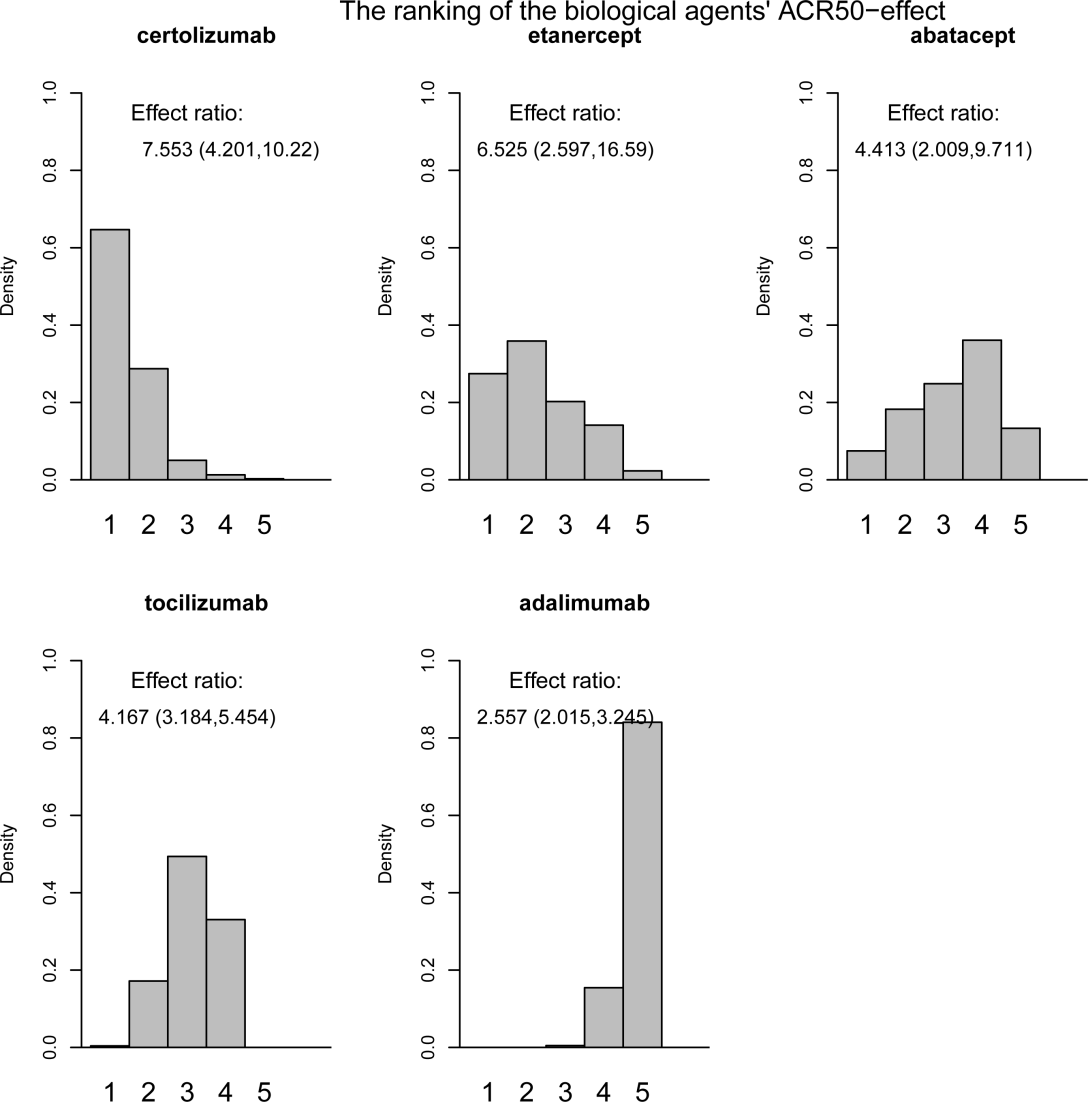
Histograms and ranking I. ACR50 effects as the response ratio versus placebo, drugs given alone.

**Fig 4:**
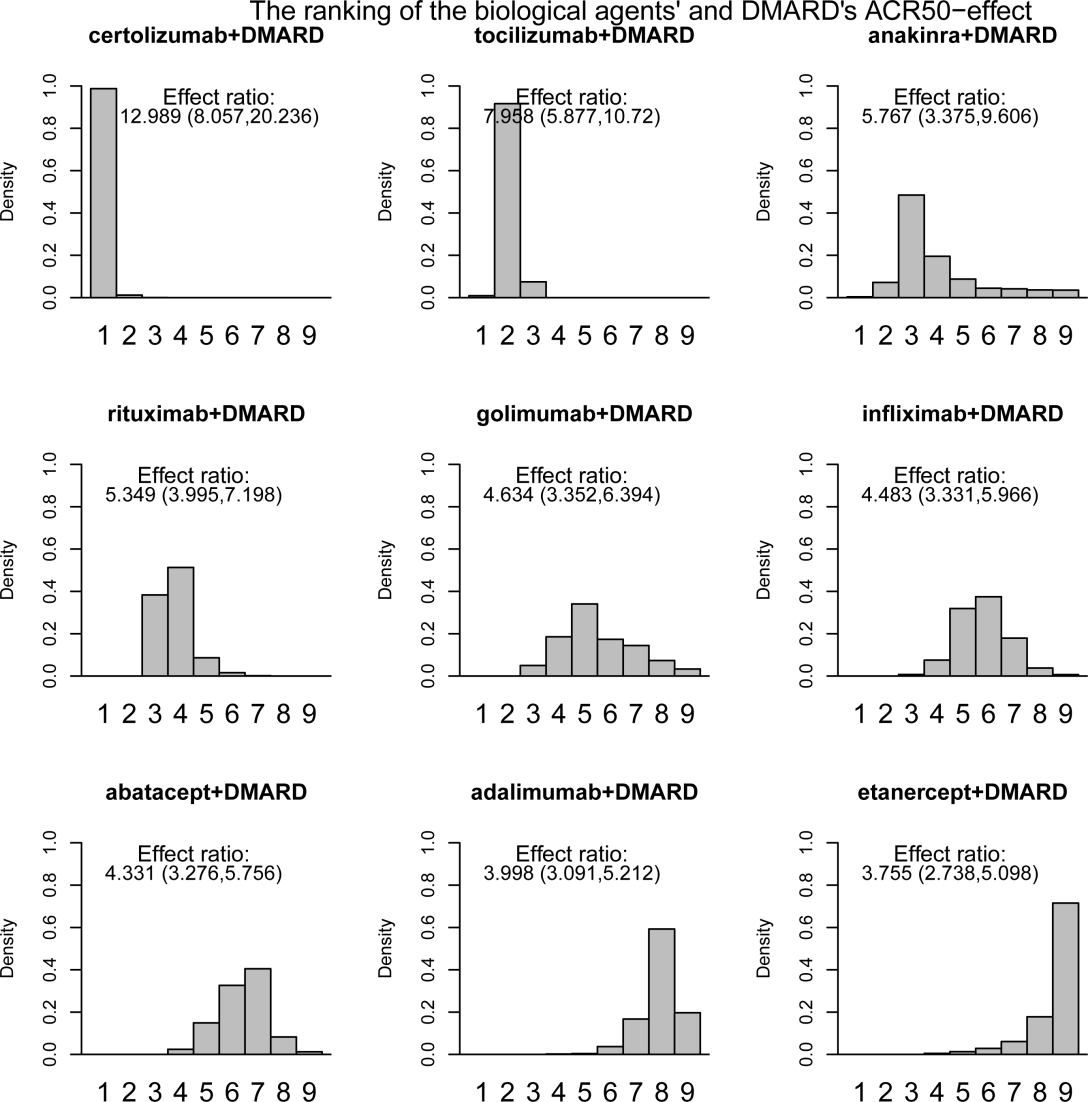
Histograms and ranking II. ACR50 effects as the response ratio versus placebo, drugs were given together with DMARD.

The ranking of the drugs when given alone was, from most to least effective, certolizumab, etanercept, tocilizumab/ abatacept and adalimumab. All drugs were however effective, with response-ratios ranging from 2.56 (credibility interval 2.02 to 3.25) for adalimumab to 7.55 (credibility interval 4.20 to 10.22) for certolizumab.

The ranking of the drugs when given together with DMARD was, from most to least effective, certolizumab, tocilizumab, anakinra/rituximab, golimumab/ infliximab/ abatacept and adalimumab/etanercept. The effect ratio of joint golimumab and DMARD versus placebo treatment was 4.63 and the corresponding effects of joint infliximab and DMARD and joint abatacept and DMARD versus placebo treatment were 4.48 and 4.33 respectively. The probabilites that joint golimumab and DMARD treatment was better than joint infliximab and DMARD and joint abatacept and DMARD treatment were 0.59 and 0.7 respectively. The probability that joint infliximab and DMARD treatment was better than joint abatacept and DMARD treatment was 0.69. Hence, one could argue that these three drugs were not that different with respect to effect.

The effect ratio of joint adalimumab and DMARD versus placebo treatment was 4.00 and the corresponding effect of joint etanercept and DMARD treatment was 3.76. Also, the probability that joint adalimumab and DMARD treatment was better than joint etanercept and DMARD treatment was 0.76. These two treatments also seem to be similar with respect to effect. All agents were effective when given together with DMARD, with response-ratios ranging from 3.76 (credibility interval 2.74 to 5.1) for etanercept to 12.99 (credibility interval 8.06 to 20.24) for certolizumab.

The ranking of drugs given jointly with DMARD was for certolizumab, abatacept and adalimumab the same as the ranking when drugs were given without DMARD treatment. Etanercept was on the other hand ranked higher and tocilizumab lower when given alone compared to given with DMARD treatment. Tocilizumab had an almost twice as high response ratio while the effect of etanercept was almost halved when given with DMARD. Hence, unlike the other drugs, etanercept had a much higher effect when given alone than with joint DMARD treatment, but the uncertainty concerning the effect of exclusive etanercept treatment was very large.

## Supporting Information

S1 File (DOCX)

## References

[1] Tvete IF, Natvig B, Gåsemyr J, Meland N, Røine M, Klemp M (2015) Comparing Effects of Biologic Agents in Treating Patients with Rheumatoid Arthritis: A Multiple Treatment Comparison Regression Analysis. PLoS ONE 10(9): e0137258 doi:10.1371/journal.pone.0137258 26356639 10.1371/journal.pone.0137258 PMC4565694

