# Supplementary material for: Comparing Effects of Biologic Agents in Treating Patients with Rheumatoid Arthritis: A Multiple Treatment Comparison Regression Analysis
Source: PLoS One. 2015 Sep 10;10(9):e0137258. doi: 10.1371/journal.pone.0137258 (PMC4565694; doi:10.1371/journal.pone.0137258)
Supplement: S1 File — (DOCX) [file pone.0137258.s002.docx]

**Supplementary material**

There were altogether 111 comparisons. Counting treatment arms, see Table 1, gave 55+29+8+5+2+1=100 comparisons. The additional 11 comparisons stems from studies with different treatment arms (excepting DMARD and placebo treatment); study 4 (9 extra comparisons), study 22 (1 extra comparison) and study 53 (one extra comparison), see Table 1.

The Supplemental Table 1 contains a list of the excluded publications and reasons for the exclusions. The references are listed at the end of this document.

| Table A. Exclusions. List of excluded publications and reasons for exclusions. | | |
| --- | --- | --- |
|  | Reference | Reason for exclusion |
| 1 | Bao 2001 | Not a double blind study |
| 2 | Bathon 2000 | Only ACR50 response given in figure |
| 3 | Blumenauer 2003 | No randomized controlled trial |
| 4 | Breedveld 2005 | No randomized controlled trial |
| 5 | Bresnihan 1998 | Lacked ACR50 response |
| 6 | Choy 2002 | Wrong comparator |
| 7 | Cohen 2011 | Lacked ACR50 response |
| 8 | Emery 2011 | Lacked ACR50 response |
| 9 | Emery 2006a | Lacked ACR50 response |
| 10 | DeFilippis 2006 | Only ACR50 response given in figure |
| 11 | Fleishmann2003 | Lacked ACR50 response |
| 12 | Geborek 2002 | No randomized controlled trial |
| 13 | Genovese 2002 | 2 year follow-up of Bathon 2000 |
| 14 | Genovese2004 | Wrong comparator |
| 15 | Genovese2005b | Lacked ACR50 response |
| 16 | Genovese 2008b | 2 years follow-up of Genovese 2005a which is included |
| 17 | Genovese 2012 | 5 years follow-up of Genovese 2005a |
| 18 | Lund Hetland2010 | No randomized controlled trial |
| 19 | Hyrich2006 | No randomized controlled trial |
| 20 | Kavanaugh 2009 | Lacked ACR50 response |
| 21 | Keystone2008b | Lacked ACR50 response |
| 22 | Keystone 2009b | Lacked ACR50 response |
| 23 | Keystone 2012 | 5 years follow-up of Cohen 2006 which is included |
| 24 | Keystone 2014 | Lacked ACR50 response |
| 25 | Kievit2008 | No randomized controlled trial |
| 26 | Kristensen2006 | No randomized controlled trial |
| 27 | Kremer2003 | Longer follow-up of Kremer 2005 which is included |
| 28 | Mathias2000 | Lacked ACR50 response |
| 29 | Moreland2002 | Only ACR50 response given in figure |
| 30 | Moreland2001 | Lacked ACR50 response |
| 31 | Russel2007 | Lacked ACR50 response |
| 32 | Schiff2009 | No randomized controlled trial |
| 33 | van der Heide2005 | Lacked ACR50 response |
| 34 | Van der Heide 2006 | 2 year follow-up of Klareskog 2004 which is included |
| 35 | Vojvodich 2007 | Children |
| 36 | Weaver 2006 | No randomized controlled trial |
| 37 | Weinblatt 2006 | Open study of adalimumab, continuation of Weinblatt2003 |
| 38 | Schiff 2014 | 2 years follow-up of Weinblatt 2013 which is included |
| 39 | Westhovens2006a | Lacked ACR50 response |

The Supplemental Table 2 contains the estimates parameters in a model with dose level as explanatory variable, where the effect of dose is independent of which TNF-inhibitor drug was given.

| **Table B. Parameter estimates.** Estimates with uncertainty, model with dose level (effect of dose independent of TNF-inhibitor choice). | | | | | | |
| --- | --- | --- | --- | --- | --- | --- |
| γ [1:6]* | 7.554 (4.364,10.23) | 2.541 (2.018,3.214) | 6.693 (2.642,17.56) | 4.138 (3.145,5.406) | 4.416 (1.942,9.62) | 2.326 (1.808,2.996) |
| γ [7:12] | 4.431 (3.338,5.739) | 4.281 (3.247,5.563) | 7.058 (4.271,11.531) | 10.988 (7.307,16.42) | 4.605 (3.276,6.331) | 3.957 (3.07,5.081) |
| γ [13:16] | 7.882 (5.813,10.465) | 5.292 (3.962,7.053) | 3.709 (2.74,5.05) |  |  |  |
| β_D_ | 0.392 (0.289,0.469) |  |  |  |  |  |
| σ_βD_ | 4.95 (0.26,18.525) |  |  |  |  |  |
| σ_φ_ | 1.783 (1.194,2.722) |  |  |  |  |  |
| *γ[1]= γCER,p, γ[2]= γADA,p, γ[3]= γETN,p, γ[4]= γTOC,p, γ[5]= γABA,p, γ[6]= γDM/DM+P,p, γ[7]= γINF+DM,p, γ[8]= γABA+DM,p, γ[9]= γANA+DM,p, γ[10]= γCER+DM,p, γ[11]= γGOL+DM,p, γ[12]= γADA+DM,p, γ[13]= γTOC+DM,p, γ[14]= γRIT+DM,p og γ[15]= γETN+DM,p. | | | | | | |

| **Table C: Patient data: responses.** Number with ACR50 scores (number of patients) for the comparator and treatment groups. | | | | | | | | |
| --- | --- | --- | --- | --- | --- | --- | --- | --- |
|  | Study | Treat_arm 1_ | Treat_arm 2_ | Treat_arm 3_ | Treat_arm 4_ | Treat_arm 5_ | Treat_arm 6_ | Treat_arm 7_ |
| 1 | Abe 2006 | 4 (47) | 18 (51) | 15 (49) |  |  |  |  |
| 2 | Emery 2008a | 6 (160) | 49 (175) | 27 (163) |  |  |  |  |
| 3 | Genovese 2008a | 37 (413) | 302 (803) |  |  |  |  |  |
| 4 | Maini2006 | 14 (49) | 3 (53) | 15 (54) | 21 (52) | 17 (52) | 18 (49) | 27 (50) |
| 5 | Smolen2008 | 22 (204) | 67 (213) | 90 (205) |  |  |  |  |
| 6 | Chen 2009 | 2 (12) | 12 (35) |  |  |  |  |  |
| 7 | Cohen 2002 | 2 (48) | 8 (63) | 9 (46) | 6 (55) | 14 (59) | 8 (46) |  |
| 8 | Cohen 2004 | 20 (251) | 43 (250) |  |  |  |  |  |
| 9 | Edwards 2004 | 5 (40) | 17 (40) |  |  |  |  |  |
| 10 | Emery 2006b | 19 (149) | 41 (124) | 65 (192) |  |  |  |  |
| 11 | Emery 2009 | 47 (160) | 64 (159) | 48 (159) |  |  |  |  |
| 12 | Emery 2010 | 16 (172) | 44 (167) | 44 (170) |  |  |  |  |
| 13 | Fleischmann 2009 | 4 (109) | 25 (111) |  |  |  |  |  |
| 14 | Kremer 2006 | 40 (219) | 209 (433) |  |  |  |  |  |
| 15 | Lipsk y2000 | 7 (88) | 18 (86) | 29 (86) | 34 (87) | 31 (81) |  |  |
| 16 | Maini 1999 | 4 (88) | 22 (86) | 25 (86) | 26 (87) | 21 (81) |  |  |
| 17 | Miyasaka 2008 | 5 (87) | 14 (87) | 22 (91) | 28 (87) |  |  |  |
| 18 | Moreland 1999 | 4 (80) | 18 (76) | 31 (78) |  |  |  |  |
| 19 | Putte 2003 | 1 (70) | 17 (72) | 19 (70) | 14 (72) |  |  |  |
| 20 | Putte 2004 | 9 (110) | 20 (106) | 23 (112) | 25 (113) | 36 (103) |  |  |
| 21 | Quinn 2005 | 4 (10) | 8 (10) |  |  |  |  |  |
| 22 | Schiff 2008 | 22 (110) | 63 (156) | 61 (165) |  |  |  |  |
| 23 | Schiff 2008 | 60 (165) | 121 (266) |  |  |  |  |  |
| 24 | Nishimoto 2004 | 1 (53) | 14 (54) | 22 (55) |  |  |  |  |
| 25 | Nishimoto 2007 | 19 (145) | 100 (157) |  |  |  |  |  |
| 26 | Nishimoto 2009 | 7 (64) | 30 (61) |  |  |  |  |  |
| 27 | Smolen 2009 | 4 (127) | 80 (246) | 81 (246) |  |  |  |  |
| 28 | StClair 2004 | 88 (282) | 160 (359) | 179 (363) |  |  |  |  |
| 29 | Weinblatt 1999 | 1 (30) | 23 (59) |  |  |  |  |  |
| 30 | Weinblatt 2003 | 5 (62) | 22 (69) | 37 (67) | 31 (73) |  |  |  |
| 31 | Westhovens 2006b | 33 (363) | 110 (360) | 119 (361) |  |  |  |  |
| 32 | Zhang 2006 | 22 (86) | 38 (87) |  |  |  |  |  |
| 33 | Furst 2003 | 34 (318) | 90 (318) |  |  |  |  |  |
| 34 | Genovese 2005a | 6 (133) | 52 (258) |  |  |  |  |  |
| 35 | Kay 2008 | 2 (35) | 42 (137) |  |  |  |  |  |
| 36 | Keystone 2004 | 19 (200) | 80 (212) | 86 (207) |  |  |  |  |
| 37 | Keystone 2008 | 15 (199) | 146 (393) | 156 (390) |  |  |  |  |
| 38 | Kim 2007 | 9 (63) | 28 (65) |  |  |  |  |  |
| 39 | Klareskog 2004 | 98 (228) | 159 (231) |  |  |  |  |  |
| 40 | Kremer 2005 | 24 (119) | 23 (105) | 48 (115) |  |  |  |  |
| 41 | Keystone 2009a | 18 (133) | 62 (178) |  |  |  |  |  |
| 42 | Cohen 2006 | 10 (209) | 80 (311) |  |  |  |  |  |
| 43 | Emery 2008b | 6 (158) | 27(161) | 49 (170) |  |  |  |  |
| 44 | Detert 2013 | 44 (85) | 46 (87) |  |  |  |  |  |
| 45 | Kavanaugh 2013 | 176 (517) | 268 (515) |  |  |  |  |  |
| 46 | Tak 2011 | 97 (252) | 141 (252) | 159 (251) |  |  |  |  |
| 47 | Choy 2012 | 7 (121) | 23 (126) |  |  |  |  |  |
| 48 | Kremer 2010 | 12 (129) | 24 (129) | 32 (128) |  |  |  |  |
| 49 | Tanaka 2012 | 13 (88) | 36 (86) | 42 (87) |  |  |  |  |
| 50 | Kremer 2011 | 35 (393) | 100 (399) | 127 (398) |  |  |  |  |
| 51 | Jones 2010 | 95 (284) | 127 (288) |  |  |  |  |  |
| 52 | Yazici 2012 | 23 (205) | 123 (409) |  |  |  |  |  |
| 53 | Breedveld 2006 | 111 (257) | 156(268) | 101 (274) |  |  |  |  |
| 54 | Westhovens 2009 | 147 (256) | 107 (253) |  |  |  |  |  |
| 55 | Weinblatt 2013 | 147 (318) | 151 (328) |  |  |  |  |  |

| **Table D. Patient data: disease duration.** Duration of RA at trial start for the comparator and treatment groups. | | | | | | | | |
| --- | --- | --- | --- | --- | --- | --- | --- | --- |
|  | Study | V_arm 1_ | V_arm 2_ | V_arm 3_ | V_arm 4_ | V_arm 5_ | V_arm 6_ | V_arm 7_ |
| 1 | Abe 2006 | 7.5 | 9.1 | 7.1 |  |  |  |  |
| 2 | Emery 2008a | 11.4 | 12.6 | 11 |  |  |  |  |
| 3 | Genovese 2008a | 9.8 | 9.8 |  |  |  |  |  |
| 4 | Maini2006 | 0.94 | 0.77 | 0.82 | 0.77 | 0.78 | 0.65 | 0.89 |
| 5 | Smolen2008 | 7.8 | 0.94 | 7.5 |  |  |  |  |
| 6 | Chen 2009 | 8.3 | 6.2 |  |  |  |  |  |
| 7 | Cohen 2002 | 7.8 | 6.3 | 8.8 | 7 | 6.5 | 8 |  |
| 8 | Cohen 2004 | 10 | 11 |  |  |  |  |  |
| 9 | Edwards 2004 | 11 | 9 | 12 |  |  |  |  |
| 10 | Emery 2006b | 9.3 | 11.1 | 10.8 |  |  |  |  |
| 11 | Emery 2009 | 2.9 | 3.5 | 3.6 |  |  |  |  |
| 12 | Emery 2010 | 7.48 | 7.1 | 6.61 |  |  |  |  |
| 13 | Fleischmann 2009 | 10.4 | 8.7 |  |  |  |  |  |
| 14 | Kremer 2006 | 8.9 | 8.5 |  |  |  |  |  |
| 15 | Lipsky 2000 | 11 | 10 | 9 | 11 | 12 |  |  |
| 16 | Maini 1999 | 8.9 | 8.4 | 7.2 | 9 | 8.7 |  |  |
| 17 | Miyasaka 2008 | 8.4 | 10 | 9.9 | 9.5 |  |  |  |
| 18 | Moreland 1999 | 12 | 13 | 11 |  |  |  |  |
| 19 | Putte 2003 | 9.4 | 10.4 | 10 | 10.1 |  |  |  |
| 20 | Putte 2004 | 11.6 | 9.3 | 11.3 | 10.6 | 11.9 |  |  |
| 21 | Quinn 2005 | 0.5 | 0.62 |  |  |  |  |  |
| 22 | Schiff 2008 | 8.4 | 7.9 | 7.3 |  |  |  |  |
| 23 | Schiff 2008 | 7.3 | 7.9 |  |  |  |  |  |
| 24 | Nishimoto 2004 | 8.4 | 7.3 | 8.3 |  |  |  |  |
| 25 | Nishimoto 2007 | 2.4 | 2.2 |  |  |  |  |  |
| 26 | Nishimoto 2009 | 8.7 | 8.5 |  |  |  |  |  |
| 27 | Smolen 2009 | 5.6 | 6.1 | 6.5 |  |  |  |  |
| 28 | StClair 2004 | 0.9 | 0.8 | 0.9 |  |  |  |  |
| 29 | Weinblatt 1999 | 13 | 13 |  |  |  |  |  |
| 30 | Weinblatt 2003 | 11.1 | 13.1 | 12.2 | 12.8 |  |  |  |
| 31 | Westhovens 2006b | 8.4 | 7.8 | 7.8 |  |  |  |  |
| 32 | Zhang 2006 | 8 | 7.13 |  |  |  |  |  |
| 33 | Furst 2003 | 11.5 | 9.3 |  |  |  |  |  |
| 34 | Genovese 2005a | 11.4 | 12.2 |  |  |  |  |  |
| 35 | Kay 2008 | 5.6 | 8.2 |  |  |  |  |  |
| 36 | Keystone 2004 | 10.9 | 11 | 11 |  |  |  |  |
| 37 | Keystone 2008 | 6.2 | 6.1 | 6.2 |  |  |  |  |
| 38 | Kim 2007 | 6.9 | 6.8 |  |  |  |  |  |
| 39 | Klareskog 2004 | 6.8 | 6.8 |  |  |  |  |  |
| 40 | Kremer 2005 | 8.9 | 9.7 | 9.7 |  |  |  |  |
| 41 | Keystone 2009a | 0.56 | 0.52 |  |  |  |  |  |
| 42 | Cohen 2006 | 0.13 | 0.15 |  |  |  |  |  |
| 43 | Emery 2008b | 0.38 | 0.33 |  |  |  |  |  |
| 44 | Detert 2013 | 0.56 | 0.52 |  |  |  |  |  |
| 45 | Kavanaugh 2013 | 0.13 | 0.15 |  |  |  |  |  |
| 46 | Tak 2011 | 0.38 | 0.33 | 0.92 |  |  |  |  |
| 47 | Choy 2012 | 9.9 | 9.4 |  |  |  |  |  |
| 48 | Kremer 2010 | 7.4 | 8.1 | 9.4 |  |  |  |  |
| 49 | Tanaka 2012 | 8.7 | 8.8 | 8.1 |  |  |  |  |
| 50 | Kremer 2011 | 9 | 9.4 | 9.3 |  |  |  |  |
| 51 | Jones 2010 | 6.2 | 6.4 |  |  |  |  |  |
| 52 | Yazici 2012 | 8.5 | 8.6 |  |  |  |  |  |
| 53 | Breedveld 2006 | 0.8 | 0.7 | 0.7 |  |  |  |  |
| 54 | Westhovens 2009 | 0.91 | 0.99 |  |  |  |  |  |
| 55 | Weinblatt 2013 | 1.7 | 1.9 |  |  |  |  |  |

| **Table E. Study dose levels.** Dose level of RA at trial start, the comparator and treatment groups. | | | | | | | | |
| --- | --- | --- | --- | --- | --- | --- | --- | --- |
|  | Study | D_arm 1_ | D_arm 2_ | D_arm 3_ | D_arm 4_ | D_arm 5_ | D_arm 6_ | D_arm 7_ |
| 1 | Abe 2006 |  | 1 | 2 |  |  |  |  |
| 2 | Emery 2008a |  | 2 | 1 |  |  |  |  |
| 3 | Genovese 2008a |  | 2 |  |  |  |  |  |
| 4 | Maini2006 |  | 1 | 1 | 2 | 1 | 1 | 2 |
| 5 | Smolen2008 |  | 1 | 2 |  |  |  |  |
| 6 | Chen 2009 |  | 1 |  |  |  |  |  |
| 7 | Cohen 2002 |  | 1 | 1 | 1 | 1 | 1 |  |
| 8 | Cohen 2004 |  | 2 |  |  |  |  |  |
| 9 | Edwards 2004 |  | 2 | 2 |  |  |  |  |
| 10 | Emery 2006b |  | 1 | 2 |  |  |  |  |
| 11 | Emery 2009 |  | 1 | 2 |  |  |  |  |
| 12 | Emery 2010 |  | 2 | 2 |  |  |  |  |
| 13 | Fleischmann 2009 |  | 2 |  |  |  |  |  |
| 14 | Kremer 2006 |  | 2 |  |  |  |  |  |
| 15 | Lipsky 2000 |  | 1 | 1 | 2 | 2 |  |  |
| 16 | Maini 1999 |  | 1 | 1 | 2 | 2 |  |  |
| 17 | Miyasaka 2008 |  | 1 | 1 | 2 |  |  |  |
| 18 | Moreland 1999 |  | 1 | 2 |  |  |  |  |
| 19 | Putte 2003 |  | 1 | 1 | 2 |  |  |  |
| 20 | Putte 2004 |  | 1 | 1 | 1 | 1 |  |  |
| 21 | Quinn 2005 |  | 1 |  |  |  |  |  |
| 22 | Schiff 2008 |  | 2 | 1 |  |  |  |  |
| 23 | Schiff 2008 | 1 | 2 |  |  |  |  |  |
| 24 | Nishimoto 2004 |  | 1 | 2 |  |  |  |  |
| 25 | Nishimoto 2007 |  | 2 |  |  |  |  |  |
| 26 | Nishimoto 2009 |  | 2 |  |  |  |  |  |
| 27 | Smolen 2009 |  | 2 | 2 |  |  |  |  |
| 28 | StClair 2004 |  | 1 | 1 |  |  |  |  |
| 29 | Weinblatt 1999 |  | 2 |  |  |  |  |  |
| 30 | Weinblatt 2003 |  | 1 | 1 | 2 |  |  |  |
| 31 | Westhovens 2006b |  | 1 | 2 |  |  |  |  |
| 32 | Zhang 2006 |  | 1 |  |  |  |  |  |
| 33 | Furst 2003 |  | 1 |  |  |  |  |  |
| 34 | Genovese 2005a |  | 2 |  |  |  |  |  |
| 35 | Kay 2008 |  | 2 |  |  |  |  |  |
| 36 | Keystone 2004 |  | 1 | 1 |  |  |  |  |
| 37 | Keystone 2008 |  | 1 | 2 |  |  |  |  |
| 38 | Kim 2007 |  | 1 |  |  |  |  |  |
| 39 | Klareskog 2004 |  | 2 |  |  |  |  |  |
| 40 | Kremer 2005 |  | 1 | 2 |  |  |  |  |
| 41 | Keystone 2009a |  | 2 |  |  |  |  |  |
| 42 | Cohen 2006 |  | 2 |  |  |  |  |  |
| 43 | Emery 2008b |  | 1 | 2 |  |  |  |  |
| 44 | Detert 2013 |  | 1 |  |  |  |  |  |
| 45 | Kavanaugh 2013 |  | 1 |  |  |  |  |  |
| 46 | Tak 2011 |  | 2 | 2 |  |  |  |  |
| 47 | Choy 2012 |  | 2 |  |  |  |  |  |
| 48 | Kremer 2010 |  | 1 | 2 |  |  |  |  |
| 49 | Tanaka 2012 |  | 1 | 2 |  |  |  |  |
| 50 | Kremer 2011 |  | 1 | 2 |  |  |  |  |
| 51 | Jones 2010 |  | 2 |  |  |  |  |  |
| 52 | Yazici 2012 |  | 2 |  |  |  |  |  |
| 53 | Breedveld 2006 |  | 1 | 1 |  |  |  |  |
| 54 | Westhovens 2009 |  | 2 |  |  |  |  |  |
| 55 | Weinblatt 2013 | 1 | 2 |  |  |  |  |  |

| **Table F. Actual dose levels.** Dose levels of RA at trial start, the comparator and treatment groups. | | | | | | | | |
| --- | --- | --- | --- | --- | --- | --- | --- | --- |
|  | Study | D_arm 1_ | D_arm 2_ | D_arm 3_ | D_arm 4_ | D_arm 5_ | D_arm 6_ | D_arm 7_ |
| 1 | Abe 2006 |  | 3mg/kg week026 | 10mg/kgweek026 | - | - | - | - |
| 2 | Emery 2008a |  | 8mg/kg each 4.week | 4mg/kg each 4.week | - | - | - | - |
| 3 | Genovese 2008a |  | 8mg/kg each 4.week | - | - | - | - | - |
| 4 | Maini2006 |  | 2mg/kg each 4.week | 4mg/kg each 4.week | 8mg/kg each 4.week | 2mg/kg each 4.week | 4mg/kg each 4.week | 8mg/kg each 4.week |
| 5 | Smolen2008 |  | 4mg/day every four week | 8mg/day every four week | - | - | - | - |
| 6 | Chen 2009 |  | 40mg each 2. week | - | - | - | - | - |
| 7 | Cohen 2002 |  | 0.04mg/kg day | 0.1mg/kg day | 0.4mg/kg day | 1mg/kg day | 2mg/kg day | - |
| 8 | Cohen 2004 |  | 100mg/day | - | - | - | - | - |
| 9 | Edwards 2004 |  | 1000mg day 1 and 15 | 1000mg day1and15 | - | - | - | - |
| 10 | Emery 2006b |  | 500mg day 1 and 15 | 1000mg day1and15 | - | - | - | - |
| 11 | Emery 2009 |  | 50mg each 4. week | 100mg each4week | - | - | - | - |
| 12 | Emery 2010 |  | 2*500 day1and15 | 2*1000 day 1 and 15 | - | - | - | - |
| 13 | Fleischmann 2009 |  | 400mg each 4. week | - | - | - | - | - |
| 14 | Kremer 2006 |  | 10mg/kg day 1, 15 and 29, and so each 28 day | - | - | - | - | - |
| 15 | Lipsky 2000 |  | 3mg/kg each 8.week | 3mg/kg each 4.week | 10mg/kg each 8.week | 10mg/kg each 4.week | - | - |
| 16 | Maini 1999 |  | 3mg/kg week 0, 2 and 6 and so each 8.week | 3mg/kg week 0, 2 and 6 and so each 4.week | 10mg/kg week 0, 2 and 6 and so each 8.week | 10mg/kg week 0, 2 and 6 and so each 4.week | - | - |
| 17 | Miyasaka 2008 |  | 20mg each 2.week | 40mg each 2.week | 80mg each 2.week | - | - | - |
| 18 | Moreland 1999 |  | 10mg 2 g week | 25mg 2 g week | - | - | - | - |
| 19 | Putte 2003 |  | 20mg each week | 40mg each week | 80mg each week | - | - | - |
| 20 | Putte 2004 |  | 20mg each 2.week | 20mg each week | 40mg each 2.week | 40mg each week | - | - |
| 21 | Quinn 2005 |  | 3 mg/kg day1, week 2 and 6, and so each 8.week | - | - | - | - | - |
| 22 | Schiff 2008 | placebo each 4.week | 10mg/kg each 4.week | 3mg/kg each 8.week | - | - | - | - |
| 23 | Schiff 2008 | 3mg/kg each 8.week | 10mg/kg each 4.week | - | - | - | - | - |
| 24 | Nishimoto 2004 |  | 4mg/kg each 4.week | 8mg/kg each 4. week | - | - | - | - |
| 25 | Nishimoto 2007 |  | 8mg/kg each 4. week | - | - | - | - | - |
| 26 | Nishimoto 2009 |  | 8mg/kg each 4. week | - | - | - | - | - |
| 27 | Smolen 2009 |  | 400 mg week 0, 2 and 4, and so 200mg each 2. week | 400mg week 0, 2 and 4, and so 400mg each 2.week | - | - | - | - |
| 28 | StClair 2004 |  | 3mg/kg week 0, 2 and 6, and so each 8.week | 6mg/kg week 0, 2 and 6, and so each 8.week | - | - | - | - |
| 29 | Weinblatt 1999 |  | 25mg 2 g week | - | - | - | - | - |
| 30 | Weinblatt 2003 |  | 20mg each 2.week | 40mg each 2.week | 80mg each 2.week | - | - | - |
| 31 | Westhovens 2006b |  | 3mg/kg week 0, 2, 6 and 14 | 10mg/kg week 0, 2, 6 and 14 | - | - | - | - |
| 32 | Zhang 2006 |  | 3mg/kg week 0, 2, 6 and 14 | - | - | - | - | - |
| 33 | Furst 2003 |  | 40mgeveryotherweek | - | - | - | - | - |
| 34 | Genovese 2005a |  | 10 mg/kg day 1, 15 and 29, and so each 28 day | - | - | - | - | - |
| 35 | Kay 2008 |  | 100mgeach4week | - | - | - | - | - |
| 36 | Keystone 2004 |  | 20mg each 2.week | 40mg each 2.week | - | - | - | - |
| 37 | Keystone 2008 |  | 200mg each 2.week | 400mg each 2.week | - | - | - | - |
| 38 | Kim 2007 |  | 40mg each 2.week | - | - | - | - | - |
| 39 | Klareskog 2004 |  | 25mg 2 g week | - | - | - | - | - |
| 40 | Kremer 2005 |  | 2mg/kg each 4.week | 10mg/kg each 4.week | - | - | - | - |
| 41 | Keystone 2009a |  | 100mg each 4^.^ week |  |  |  |  |  |
| 42 | Cohen 2006 |  | 2*1000 day 1 and 15 |  |  |  |  |  |
| 43 | Emery 2008b |  | 4mg/kg each 4.week | 8mg/kg each 4. week |  |  |  |  |
| 44 | Detert 2013 |  | 40mg each 2.week |  |  |  |  |  |
| 45 | Kavanaugh 2013 |  | 40mg each 2.week |  |  |  |  |  |
| 46 | Tak 2011 |  | 2*500 day 1 and 15 | 2*1000 day 1 and 15 |  |  |  |  |
| 47 | Choy 2012 |  | 400mg each 4.week |  |  |  |  |  |
| 48 | Kremer 2010 |  | 2mg/kg each 12.week | 4mg/kg each 12.week |  |  |  |  |
| 49 | Tanaka 2012 |  | 50mg each 4. week | 100mgeach4week |  |  |  |  |
| 40 | Kremer 2011 |  | 4mg/kg each 4. week | 8mg/kg each 4. week |  |  |  |  |
| 51 | Jones 2010 |  | 8mg/kg each 4. week |  |  |  |  |  |
| 52 | Yazici 2012 |  | 8mg/kg each 4. week |  |  |  |  |  |
| 53 | Breedveld 2006 |  | 40mg each 2.week | 40mg each 2.week |  |  |  |  |
| 54 | Westhovens 2009 |  | 10mg/kg each 4.week |  |  |  |  |  |
| 55 | Weinblatt 2013 | 40mg each 2. week | 125mg/week |  |  |  |  |  |
| *Some patients increased the dose to 40 mg each week after week 16 in the trial | | | | | | | | |

**References, excluded publications**

1. Bao J, Yue T, Liu W, Zhang Q, Zhou L, Xu HJ, Dai SM. Secondary failure to treatment with recombinant human IL-1 receptor antagonist in Chinese patients with rheumatoid Arthritis. Clin Rheumatol 2011; 30:697–701.
2. Bathon JM, Martin RW, Fleischmann RM, Tesser JR, Schiff MH, Keystone EC, Genovese MC, Wasko MC, Moreland LW, Weaver AL, Markenson J, Finck BK. A comparison of etanercept and methotrexate in patients with early rheumatoid arthritis. N Engl J Med 2000; 343:1586-93.
3. Blumenauer A, Coyle D, Hochberg MC, Tugwell P, Wells GA. Etanercept for the treatment of rheumatoid arthritis. Cochrane Database of Systematic Reviews 2003, Issue 3. Art. No.: CD004525. DOI: 10.1002/14651858.CD004525.
4. Breedveld FC, Han C, Bala M, van der Heijde D, Baker D, Kavanaugh AF, Maini RN, Lipsky PE. Association between baseline radiographic damage and improvement in physical function after treatment of patients with rheumatoid arthritis. Ann Rheum Dis 2005; 64:52–55.
5. Bresnihan B, Alvaro-Gracia JM, Cobby M, Doherty M, Domljan Z, Emery P, Nuki G, Pavelka K, Rau R, Rozman B, Watt I, Williams B, Aitchison R, McCabe D, Musikic P. Treatment of rheumatoid arthritis with recombinant human interleukin-1 receptor antagonist. Arthritis Rheum. 1998 Dec;41(12):2196-204
6. Choy EHS, Hazleman B, Smith M, Moss K, Lisi L, Scott DGI, Patel J, Sopwith M, Isenberg DA. Efficacy of a novel PEGylated humanized anti-TNF fragment (CDP870) in patients with rheumatoid arthritis: a phase II double-blinded, randomized, dose-escalating trial. Rheumatology 2002; 41:1133–1137.
7. Cohen SB, Potter H, Deodhar A, Emery P, Conaghan P, Ostergaard M. Extremity Magnetic Resonance Imaging in Rheumatoid Arthritis: Updated Literature Review. Arthritis Care & Research Vol. 63, No. 5, May 2011, pp 660–665
8. Emery P, Fleischmann R, van der Heijde D, Keystone EC, Genovese MC, Conaghan PG, Hsia EC, Xu W, Baratelle A, Beutler A, Rahman MU. The Effects of Golimumab on Radiographic Progression in Rheumatoid Arthritis Results of Randomized Controlled Studies of Golimumab Before Methotrexate Therapy and Golimumab After Methotrexate Therapy. ARTHRITIS & RHEUMATISM Vol. 63, No. 5, May 2011, pp 1200–1210.
9. Emery P, Kosinski M, Li T, Martin M, Rhys Williams G, Becker J-C, Blaisdell B, Ware Jr JE, Birbara C, Russell AS. Treatment of Rheumatoid Arthritis Patients with Abatacept and Methotrexate Significantly Improved Health-Related Quality of Life. J Rheumatol 2006a; 33; 681-689.
10. De Filippis L, Caliri A, Anghelone S, Scibilia G, Lo Gullo R, Bagnato G. Improving outcomes in tumour necrosis factor a treatment: comparison of the efficacy of the tumour necrosis factor a blocking agents etanercept and infliximab in patients with active rheumatoid arthritis. Panminerva Med. 2006 Jun; 48(2):129-35.
11. Fleischmann RM, Schechtman J, Bennett R, Handel ML, Burmester GR, Tesser J, Modafferi D, Poulakos J, Sun G, for the 990757 Study Group. Anakinra, a Recombinant Human Interleukin-1 Receptor Antagonist (r-metHuIL-1ra), in Patients With Rheumatoid Arthritis. A Large, International, Multicenter, Placebo-Controlled Trial. ARTHRITIS & RHEUMATISM Vol. 48, No. 4, April 2003, pp 927–934
12. Geborek P, Crnkic M, Petersson IF, Saxne T, for the South Swedish Arthritis Treatment Group. Etanercept, infliximab, and leflunomide in established rheumatoid arthritis: clinical experience using a structured follow up programme in southern Sweden. Ann Rheum Dis 2002; 61:793–798.
13. Genovese MC, Bathon JM, Martin RW, et al. Etanercept versus methotrexate in patients with early rheumatoid arthritis: two-year radiographic and clinical outcomes. Arthritis Rheum 2002; 46:1443–50.
14. Genovese MC, Cohen S, Moreland L, Lium D, Robbins S, Newmark R, Bekker P, for the 20000223 Study Group. Combination Therapy With Etanercept and Anakinra in the Treatment of Patients With Rheumatoid Arthritis Who Have Been Treated Unsuccessfully With Methotrexate. ARTHRITIS & RHEUMATISM Vol. 50, No. 5, May 2004, 1412–19.
15. Genovese MC, Joan M Bathon, Roy M Fleischmann, Larry W Moreland, Richard W Martin, James B Whitmore, Wayne H Tsuji and Jonathan A Leff. Longterm safety, efficacy, and radiographic outcome with etanercept treatment in patients with early rheumatoid arthritis. J Rheumatol 2005b; 32; 1232-1242.
16. Genovese MC, Schiff M, Luggen M, Becker J-C, Aranda R, Teng J, Li T, Schmidely N, Le Bars M, Dougados M. Efficacy and safety of the selective co-stimulation modulator abatacept following 2 years of treatment in patients with rheumatoid arthritis and an inadequate response to anti-tumour necrosis factor therapy. Ann Rheum Dis 2008b; 67:547–554.
17. [Genovese MC](http://www.ncbi.nlm.nih.gov.proxy.helsebiblioteket.no/pubmed/?term=Genovese%20MC%5BAuthor%5D&cauthor=true&cauthor_uid=22798265), [Schiff M](http://www.ncbi.nlm.nih.gov.proxy.helsebiblioteket.no/pubmed/?term=Schiff%20M%5BAuthor%5D&cauthor=true&cauthor_uid=22798265), [Luggen M](http://www.ncbi.nlm.nih.gov.proxy.helsebiblioteket.no/pubmed/?term=Luggen%20M%5BAuthor%5D&cauthor=true&cauthor_uid=22798265), [Le Bars M](http://www.ncbi.nlm.nih.gov.proxy.helsebiblioteket.no/pubmed/?term=Le%20Bars%20M%5BAuthor%5D&cauthor=true&cauthor_uid=22798265), [Aranda R](http://www.ncbi.nlm.nih.gov.proxy.helsebiblioteket.no/pubmed/?term=Aranda%20R%5BAuthor%5D&cauthor=true&cauthor_uid=22798265), [Elegbe A](http://www.ncbi.nlm.nih.gov.proxy.helsebiblioteket.no/pubmed/?term=Elegbe%20A%5BAuthor%5D&cauthor=true&cauthor_uid=22798265), [Dougados M](http://www.ncbi.nlm.nih.gov.proxy.helsebiblioteket.no/pubmed/?term=Dougados%20M%5BAuthor%5D&cauthor=true&cauthor_uid=22798265). Longterm safety and efficacy of abatacept through 5 years of treatment in patients with rheumatoid arthritis and an inadequate response to tumor necrosis factor inhibitor therapy. [J Rheumatol](http://www.ncbi.nlm.nih.gov.proxy.helsebiblioteket.no/pubmed/?term=Genovese+2012+and+abatacept) 2012 Aug; 39(8):1546-54.
18. Hetland ML, Christensen IJ, Tarp U, Dreyer L, Hansen A, Hansen IT, Kollerup G, Linde L, Lindegaard HM, Poulsen UE, Schlemmer A, Jensen DV, Jensen S, Hostenkamp G, Østergaard M. Direct Comparison of Treatment Responses, Remission Rates, and Drug Adherence in Patients With Rheumatoid Arthritis Treated With Adalimumab, Etanercept, or Infliximab. Arthritis Rheum 2010 Jan; 62(1):22-32.
19. Hyrich KL, Symmons DPM, Watson KD, Silman AJ, on behalf of the British Society for Rheumatology Biologics Register Comparison of the Response to Infliximab or Etanercept Monotherapy With the Response to Cotherapy With Methotrexate or Another Disease-Modifying Antirheumatic Drug in Patients With Rheumatoid Arthritis. ARTHRITIS & RHEUMATISM Vol. 54, No. 6, June 2006, pp 1786–1794.
20. Kavanaugh A, Smolen JS, Emery P, Purcaru O, Keystone E, Richard L, Strand V, Van Vollenhoven RF. Effect of Certolizumab Pegol With Methotrexate on Home and Work Place Productivity and Social Activities in Patients With Active Rheumatoid Arthritis. Arthritis & Rheumatism (Arthritis Care & Research) Vol. 61, No. 11, November 15, 2009, pp 1592–1600
21. Keystone EC, Burmester GR, Furie R, Loveless JE, Emery P, Kremer J, Tak PP, Broder MS, Yu E, Cravets M, Magrini F, Jost F. Improvement in Patient-Reported Outcomes in a Rituximab Trial in Patients With Severe Rheumatoid Arthritis Refractory to Anti–Tumor Necrosis Factor Therapy. Arthritis & Rheumatism (Arthritis Care & Research) Vol. 59, No. 6, June 15, 2008b, 785–793.
22. Keystone E, P Emery, C G Peterfy, P P Tak, S Cohen, M C Genovese, M Dougados, G R Burmester, M Greenwald, T K Kvien, S Williams, D Hagerty, M W Cravets, T Shaw. Rituximab inhibits structural joint damage in patients with rheumatoid arthritis with an inadequate response to tumour necrosis factor inhibitor therapies. Ann Rheum Dis 2009b; 68:216–221.
23. Keystone EC, Cohen SB, Emery P, Kremer JM, Dougados M, Loveless JE, Chung C, Wong P, Lehane PB, Tyrrell H. Multiple Courses of Rituximab Produce Sustained Clinical and Radiographic Efficacy and Safety in Patients with Rheumatoid Arthritis and an Inadequate Response to 1 or More Tumor Necrosis Factor Inhibitors: 5-Year Data from the REFLEX Study. J Rheumatol 2012; 39; 2238-2246.
24. Keystone EC, Anisfeld A, Ogale S, Devenport JN, Curtis JR. Continued Benefit of Tocilizumab Plus Disease-modifying Antirheumatic Drug Therapy in Patients with Rheumatoid Arthritis and Inadequate Clinical Responses by Week 8 of Treatment. J Rheumatol 2014; 41;216-226.
25. Kievit W, Adang EM, Fransen J, Kuper HH, van de Laar MAFJ, Jansen TL, De Gendt CMA, De Rooij DJRAM, Brus HLM, Van Oijen PCM, Van Riel PCLM. The effectiveness and medication costs of three anti-tumour necrosis factor a agents in the treatment of rheumatoid arthritis from prospective clinical practice data. Ann Rheum Dis 2008; 67:1229–1234.
26. Kristensen LE, Saxne T, Geborek P. The LUNDEX, a New Index of Drug Efficacy in Clinical Practice Results of a Five-Year Observational Study of Treatment With Infliximab and Etanercept Among Rheumatoid Arthritis Patients in Southern Sweden. ARTHRITIS & RHEUMATISM. Vol. 54, No. 2, February 2006, 600–606.
27. Kremer JM, Westhovens R, Leon M, Di Giorgio E, Alten R, Steinfeld S, Russell A, Dougados M, Emery P, Nuamah IF, Williams GR, Becker JC, Hagerty DT, Moreland LW. Treatment of Rheumatoid Arthritis by Selective Inhibition of T-Cell Activation with Fusion Protein CTLA4Ig. N Engl J Med 2003; 349:1907-15.
28. Mathias SD, Colwell HH, Miller DI, Moreland LW, Buatti M, Wanke L. Health-Related Quality of Life and Functional Status of Patients with Rheumatoid Arthritis Randomly Assigned to Receive Etanercept or Placebo. Clinicaltherapeutics.22, No. 1,2000, 128-39.
29. Larry W. Moreland, Stanley B. Cohen, Scott W. Baumgartner, Elizabeth A. Tindall, Ken Bulpitt, Richard Martin, Michael Weinblatt, James Taborn, Arthur Weaver, Daniel J. Burge, And Michael H. Schiff. Longterm Safety and Efficacy of Etanercept in Patients with Rheumatoid ArthritisJ Rheumatol 2001; 28; 1238-44.
30. Moreland LW, Alten R, Van den Bosch F, Appelboom T, Leon M, Emery P, Cohen S, Luggen M, Shergy W, Nuamah I, Becker JC. Costimulatory blockade in patients with rheumatoid arthritis: a pilot, dose-finding, double-blind, placebo-controlled clinical trial evaluating CTLA-4Ig and LEA29Y eighty-five days after the first infusion. Arthritis Rheum. 2002 Jun; 46(6):1470-9.
31. Russell AS, Wallenstein GV, Li T, Martin MC, Maclean R, Blaisdell B, Gajria K, Cole JC, Becker JC, Emery P. Abatacept improves both the physical and mental health of patients with rheumatoid arthritis who have inadequate response to methotrexate treatment. Ann Rheum Dis 2007; 66:189–94.
32. Schiff M, Pritchard C, Huffstutter JE, Rodriguez-Valverde V, Durez P, Zhou X, Li T,Bahrt K, Kelly S, Le Bars M, Genovese MC. The 6-month safety and efficacy of abatacept inpatients with rheumatoid arthritis who underwent a washout after anti-tumour necrosis factor therapy or were directly switched to abatacept: the ARRIVE trial. Ann Rheum Dis 2009; 68:1708–14.
33. Van der Heijde D, Klareskog L, Singh A, Tornero J, Melo-Gomes J, Codreanu C, Pedersen R, Freundlich B, Fatenejad S. Patient reported outcomes in a trial of combination therapy with etanercept and methotrexate for rheumatoid arthritis: the TEMPO trial. Ann Rheum Dis 2005;65:328–334.
34. Van der Heijde D, Klareskog L, Rodriguez-Valverde V, et al. Comparison of etanercept and methotrexate, alone and combined, in the treatment of rheumatoid arthritis: two-year clinical and radiographic results from the TEMPO study, a double-blind, randomized trial. Arthritis Rheum 2006; 54:1063–74.
35. Vojvodich PF, Hansen JB, Andersson U, Sävendahl L, Hagelberg S. Etanercept Treatment Improves Longitudinal Growth in Prepubertal Children with Juvenile Idiopathic Arthritis. J Rheumatol 2007;34;2481-2485.
36. Weaver AL, Lautzenheiser RL, Schiff MH, Gibofsky A, Perruquet JL, Luetkemeyer J, Paulus HE, Xia HA, Leff JA, on behalf of the RADIUS Investigators. Real-world effectiveness of select biologic and DMARD monotherapy and combination therapy in the treatment of rheumatoid arthritis: results from the RADIUS observational registry. Current Medical Research and Opinion Vol. 22, No. , 2006, 185–198.
37. Weinblatt ME, Keystone EC, Furst DE, Kavanaugh AF, Chartash EK, Segurado OG. Long term efficacy and safety of adalimumab plus methotrexate in patients with rheumatoid arthritis: ARMADA 4 year extended study. Ann Rheum Dis 2006; 65:753–759.
38. Schiff M, Weinblatt ME, Valente R, van der Heijde D, Citera G, Elegbe A, Maldonado M, Fleischmann R. Head-to-head comparison of subcutaneous abatacept versus adalimumab for rheumatoid arthritis: two-year efficacy and safety findings from AMPLE trial. Ann Rheum Dis 2014; 73:86–94.
39. Westhovens R, Cole JC, Li T, Martin M, MacLean R, Lin P, Blaisdell B, Wallenstein GV, Aranda R, Sherrer Y. Improved health-related quality of life for rheumatoid arthritis patients treated with abatacept who have inadequate response to anti-TNF therapy in a double-blind, placebo-controlled, multicentre randomized clinical trial. Rheumatology 2006a; 45:1238–1246.
